# Rational Design of Berberine-Based FtsZ Inhibitors with Broad-Spectrum Antibacterial Activity

**DOI:** 10.1371/journal.pone.0097514

**Published:** 2014-05-13

**Authors:** Ning Sun, Fung-Yi Chan, Yu-Jing Lu, Marco A. C. Neves, Hok-Kiu Lui, Yong Wang, Ka-Yan Chow, Kin-Fai Chan, Siu-Cheong Yan, Yun-Chung Leung, Ruben Abagyan, Tak-Hang Chan, Kwok-Yin Wong

**Affiliations:** 1 Department of Applied Biology and Chemical Technology and the State Key Laboratory of Chirosciences, The Hong Kong Polytechnic University, Kowloon, Hong Kong, China; 2 School of Chemical Engineering and Light Industry, Guangdong University of Technology, Guangzhou, China; 3 Skaggs School of Pharmacy & Pharmaceutical Sciences, University of California San Diego, La Jolla, California, United States of America; 4 Molsoft L.L.C, San Diego, California, United States of America; University of Groningen, Groningen Institute for Biomolecular Sciences and Biotechnology, Netherlands

## Abstract

Inhibition of the functional activity of Filamenting temperature-sensitive mutant Z (FtsZ) protein, an essential and highly conserved bacterial cytokinesis protein, is a promising approach for the development of a new class of antibacterial agents. Berberine, a benzylisoquinoline alkaloid widely used in traditional Chinese and native American medicines for its antimicrobial properties, has been recently reported to inhibit FtsZ. Using a combination of *in silico* structure-based design and *in vitro* biological assays, 9-phenoxyalkyl berberine derivatives were identified as potent FtsZ inhibitors. Compared to the parent compound berberine, the derivatives showed a significant enhancement of antibacterial activity against clinically relevant bacteria, and an improved potency against the GTPase activity and polymerization of FtsZ. The most potent compound **2** strongly inhibited the proliferation of Gram-positive bacteria, including methicillin-resistant *S. aureus* and vancomycin-resistant *E. faecium*, with MIC values between 2 and 4 µg/mL, and was active against the Gram-negative *E. coli* and *K. pneumoniae*, with MIC values of 32 and 64 µg/mL respectively. The compound perturbed the formation of cytokinetic Z-ring in *E. coli*. Also, the compound interfered with *in vitro* polymerization of *S. aureus* FtsZ. Taken together, the chemical modification of berberine with 9-phenoxyalkyl substituent groups greatly improved the antibacterial activity via targeting FtsZ.

## Introduction

Antibiotic resistance is an alarming health problem worldwide. Antibiotic misuse creates selective pressure for survival of resistant bacterial strains and, consequently, many clinically used antibiotics such as β-lactams, aminoglycosides, tetracyclines and sulfonamides, are becoming ineffective against antibiotic-resistant bacteria [1], [2]. Infections associated with methicillin-resistant *Staphylococcus aureus* (MRSA) and vancomycin-resistant *Enterococcus faecium* (VREF) have resulted in increasing nosocomial health concerns for both patients and medical professionals [3], [4]. Thus, there is an urgent need for new antibacterial agents with innovative mechanisms of action.

Filamenting temperature-sensitive mutant Z (FtsZ), an analogue of eukaryotic tubulin, is an essential and highly conserved bacterial cytokinesis protein [5]. During bacterial cell division, FtsZ monomers self-assemble into a Z-ring, a highly dynamic cytoskeleton scaffold generated at the site of septum formation [6], [7]. The mechanism regulating assembly and organization of FtsZ into a ring-like structure involves GTP binding and hydrolysis, modulated by the interaction of the N-terminal nucleotide binding domain of one FtsZ monomer with the C-terminal GTPase-activating domain (T7-loop) on the adjacent FtsZ monomer [8]. Subsequently, FtsZ recruits other proteins to form a cell-division complex known as the divisome. Once the divisome is fully assembled, bacterial cell division is achieved by coordinated constriction and splitting of the daughter cells [9], [10].

**Figure 1:**
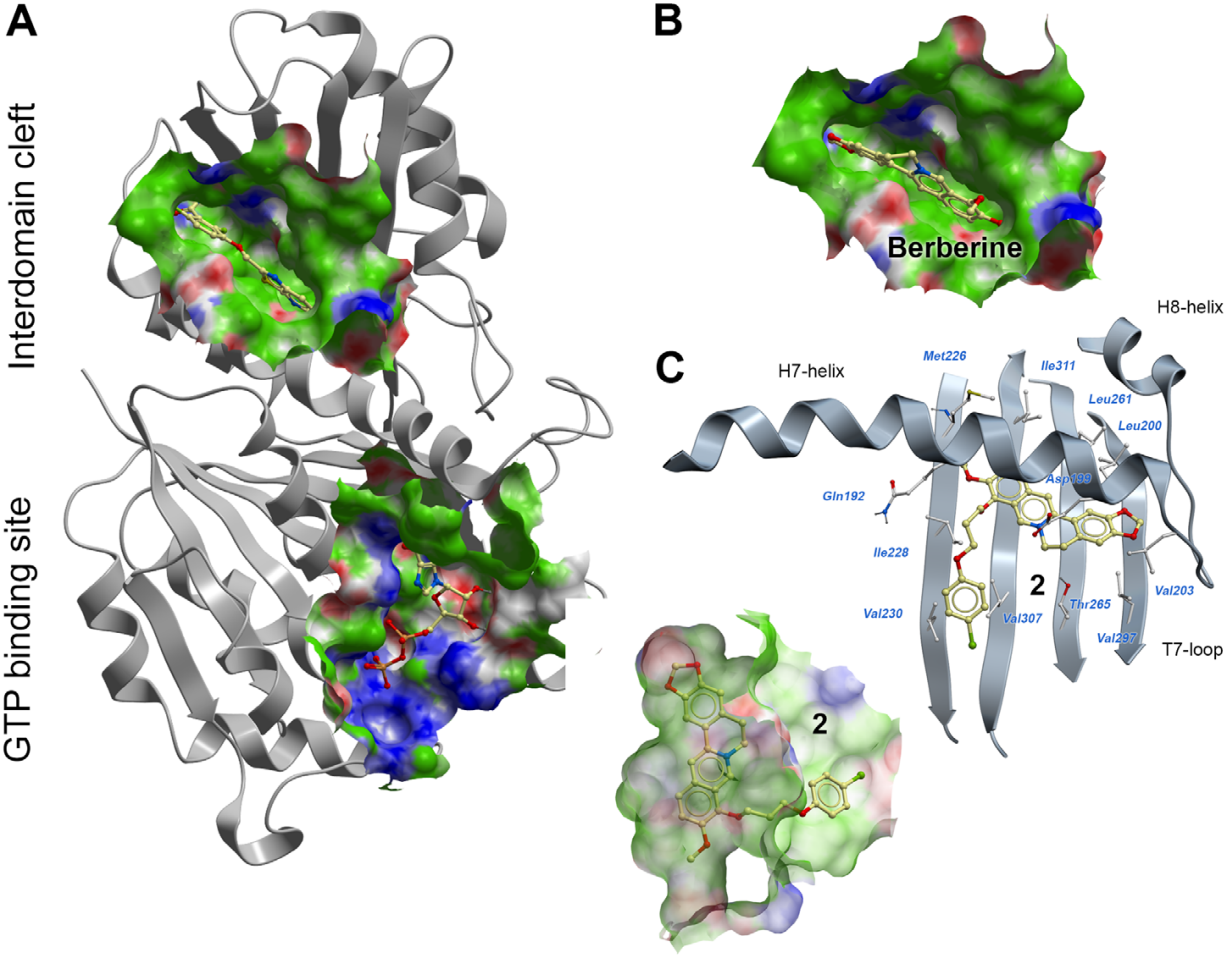
Predicted binding modes of berberine and 9-phenoxyalkyl substituted derivatives. (A) *S. aureus* FtsZ (PDB code: 4DXD) with PC190723 bound to the C-terminal interdomain cleft, and GDP bound to the N-terminal GTP binding site. (B) Predicted binding mode of berberine into the C-terminal interdomain cleft. (C) Predicted binding mode of compound **2** into the C-terminal interdomain cleft. The FtsZ pockets are colored by binding properties (white  =  neutral surface, green  =  hydrophobic surface, red  =  hydrogen bonding acceptor potential, and blue  =  hydrogen bond donor potential. Ligand atoms are shown with a ‘ball and sticks’ representation and colored in yellow (carbon), red (oxygen), blue (nitrogen), orange (phosphorus) and green (chloride). Interacting FtsZ residues are labeled and shown with white carbons.

Recent studies suggest that inhibition of bacterial cell division proteins with an essential role in bacterial cytokinesis, such as FtsZ, is a promising approach against antibiotic-resistant bacterial infections [11]–[13]. A number of small molecule inhibitors of FtsZ have already been shown to prevent FtsZ polymerization and inhibit bacterial cell division [14]–[20]. The molecules bind to one of two alternative sites of FtsZ (Figure 1A): at the N-terminal GTP binding site [21]–[23], or at the C-terminal interdomain cleft [24]. Compounds targeting the highly conserved GTP binding site mimic the natural substrate of the enzyme and might have potential advantages for developing broad-spectrum antibacterial agents [25]. However, because GTP binding sites are present in a number of human proteins, GTP-mimetic compounds might have potential liabilities related to the off-target-associated activity. Thus, the C-terminal interdomain cleft formed by residues from the C-terminal β-sheet, T7-loop and H7-helix, offers an alternative opportunity for the design of FtsZ inhibitors with therapeutic potential in antibiotic-resistant bacterial diseases [26].

**Figure 2:**
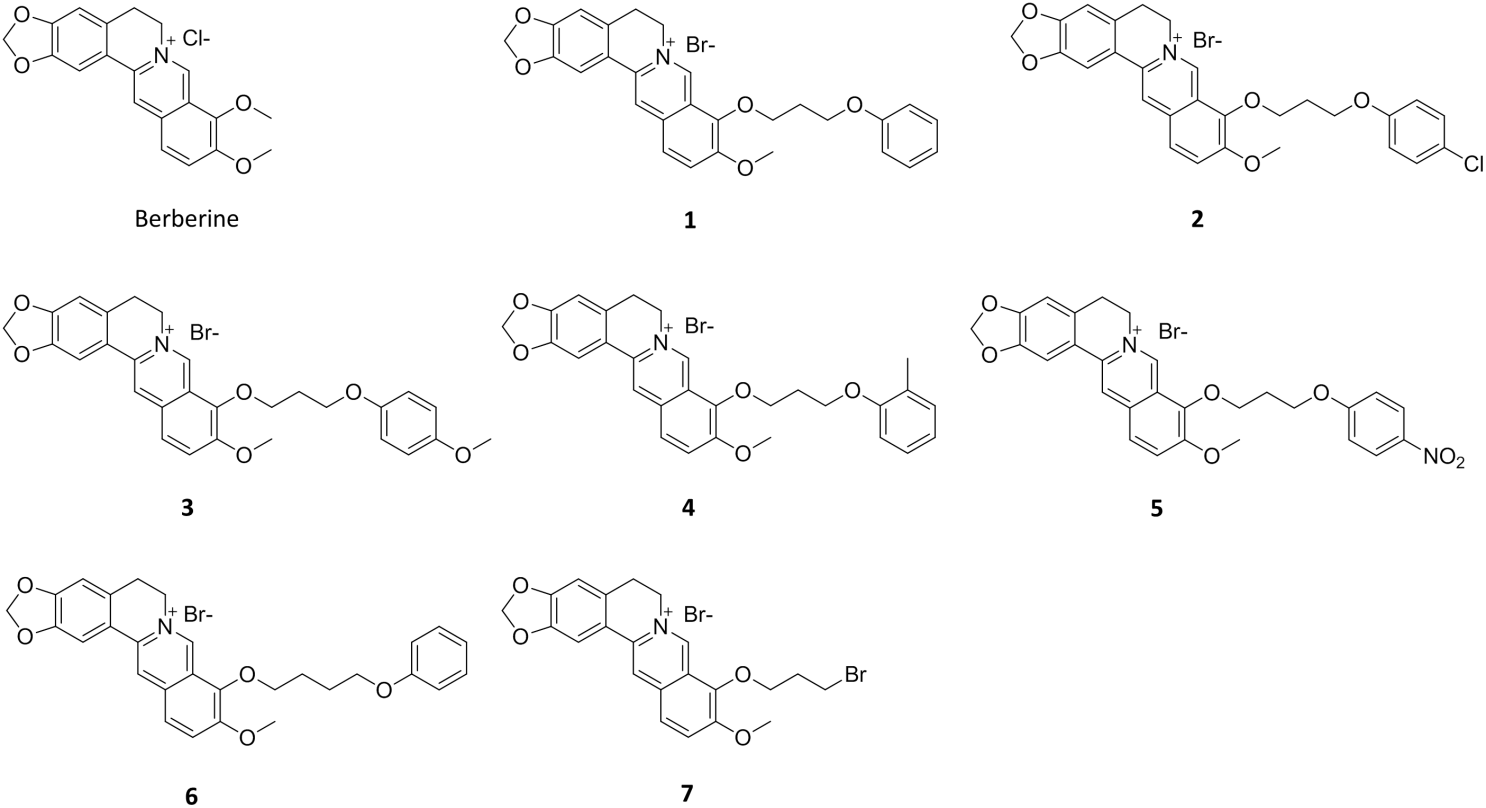
Chemical structures of berberine and its 9-phenoxyalkyl substituted derivatives.

Berberine (Figure 2) is a plant alkaloid with a long history of medicinal use in traditional Chinese and native American medicines [27]. Berberine extracts show significant antimicrobial activity against bacteria, viruses and fungi. Its potential mechanisms of antimicrobial activity include the suppression of cell adhesion and migration [28], and inhibition of microbial enzymes [29]. Moreover, recent literature reports demonstrated that berberine is active against Gram-positive bacteria with minimum inhibitory concentration values (MIC) in the range of 100–400 µg/mL by targeting the cell division protein FtsZ [30], [31]. Therefore, berberine is an attractive lead for the development of potent FtsZ inhibitors. Given the availability of X-ray crystal structures of FtsZ [7], [24], [32], [33], molecular docking is particularly appealing for guiding the chemical derivatization of berberine. Previous studies suggested that berberine binds FtsZ in a hydrophobic pocket [14]. In this paper we report the design and biological study of a series of 9-phenoxyalkyl berberine derivatives with potent inhibition of FtsZ GTPase activity and broad-spectrum of antibacterial activity.

## Materials and Methods

### Molecular Docking Simulation of Berberine and its 9-phenoxyalkyl Derivatives

The ICM molecular modeling package v3.6 was used for all computational studies. The X-ray crystal structure of the *S. aureus* FtsZ with bound GDP and PC190723 (PDB code: 4DXD) [24] was retrieved from the RCSB Protein Data Bank (http://www.rcsb.org) and prepared for docking using an automated protein preparation protocol [34]. The co-crystal ligands PC190723 and GDP were deleted before docking, as well as the water molecules. The N-terminal GTP binding site and the C-terminal interdomain cleft of *S. aureus* FtsZ were analyzed with the PocketFinder function in the ICM software [35]. Ligand binding pocket surfaces were colored according to their binding properties. Berberine was built using the ICM ligand editor, converted into 3D and minimized. The compound was docked against the N-terminal GTP binding site and the C-terminal interdomain cleft of *S. aureus* FtsZ. The sites were limited to FtsZ residues with at least one non-hydrogen atom within a 10 Å cutoff radius from the GDP and the PC190723 co-crystals, respectively. The pockets were represented by grid potential maps accounting for hydrogen bonding, hydrophobic, van der Waals, and electrostatic interactions. A grid spacing of 0.5 Å was used. Berberine was flexibly docked and scored according to the ICM scoring function. One hundred independent docking runs were performed at each site and the poses were clustered by heavy atom root-mean-square deviation (RMSD). The top-scoring results were selected and visually inspected.

A set of 9-substituted berberine derivatives (**1–7**) was docked against the X-ray crystal structure of *S. aureus* FtsZ using the same docking protocol.

### Materials and Measurement

Berberine chloride was purchased from Sigma-Aldrich. The 9-phenoxyalkyl substituted berberine derivatives (Figure 2) were synthesized according to the procedures described by Li *et al*. [36]. ^1^H NMR spectra were obtained with a Bruker 400 MHz DPX-400 NMR spectrometer. Mass spectra were recorded with a Finnigan MAT 95S mass spectrometer. All reactions were monitored by thin layer chromatography on silica gel. The synthesis of these derivatives and their ^1^H NMR and mass spectra can be referred to Text S1 in the supporting information.

### Bacterial Strains and Reagents

The bacterial strains *S. aureus* ATCC 29213, *S. aureus* ATCC 29247, *S. aureus* ATCC BAA-41, *E. faecium* ATCC 49624, *E. faecium* ATCC 700221, *E. faecalis* ATCC 29212, *S. epidermidis* ATCC 12228, *E. coli* ATCC 25922, and *K. pneumoniae* ATCC BAA-1144 used in the antimicrobial susceptibility assays were purchased from American Type Culture Collection (ATCC, USA). *B. subtilis* strain 168 was already available in our in-house collection. *E. coli* JM109 WM647 was kindly provided by Dr. W. Margolin (University of Texas-Houston Medical School). FM 4–64 was purchased from Invitrogen (Eugene, Oregon). All other chemicals and reagents were purchased from Sigma-Aldrich. Stock solutions were prepared in DMSO. The final percentage of DMSO in the assays was 1% (v/v) for all experiments.

### Expression and Purification of *S. aureus* FtsZ

*E. coli* BL21(DE3) cells were transformed with the pRSET-A-S vector carrying *S. aureus* FtsZ with a 6-histidine tag attached at its N-terminus under the control of a T7 promoter [37]. The transformed *E. coli* strain was streaked on a nutrient agar plate containing 50 µg/mL ampicillin, and the agar plate was incubated at 37°C overnight. A single colony was inoculated into 5 mL of Luria–Bertani (LB) medium in the presence of 50 µg/mL ampicillin, which was then incubated at 37°C, with shaking at 250 rpm for 16 h. The overnight culture was transferred into a fresh 2×TY medium in a dilution ratio of 1∶100 and 50 µg/mL ampicillin was then added, followed by incubation at 37°C with shaking at 250 rpm. When the OD_600_ reached 0.8, protein expression was induced with 0.4 mM isopropyl-β-D-thiogalactopyranoside (IPTG) for 4 h. Cells were harvested by centrifugation at 9000 rpm for 20 min at 4°C. The cell pellet was resuspended in 20 mL of solubilization buffer (50 mM Tris-HCl, 150 mM NaCl, 1 mM PMSF and 1 mM EDTA, pH 7.4) and then lysed with 1 mg/mL of lysozyme. The mixture was incubated for 1 h on ice. The cells were disrupted by sonication and the crude lysate obtained was centrifuged at 13,000 rpm for 1 h at 4°C. The supernatant containing 6-histidine tagged *S. aureus* FtsZ was collected and loaded onto a nickel charged HiTrap chelating column pre-equilibrated with starting buffer (20 mM sodium phosphate buffer, 0.5 M NaCl, pH 7.4). The column was then washed with 8 column volumes of the starting buffer to remove the unbound proteins, and the histidine-tagged enzyme was eluted by a linear gradient of 0−0.2 M imidazole. Fractions containing *S. aureus* FtsZ were pooled, buffer-exchanged with 20 mM NH_4_HCO_3_ (pH 8.0) at 4°C, lyophilized, and stored at −20°C. A stock solution of *S. aureus* FtsZ for the subsequent bioassay was prepared from the lyophilized powder.

### Antimicrobial Susceptibility Assays

Antimicrobial susceptibility tests were conducted in 96-well microplates using the broth microdilution procedure described in the Clinical and Laboratory Standards Institute (CLSI) guidelines [38]. Cation-adjusted Mueller Hinton broth for *S. aureus* strain ATCC 29213, methicillin-resistant *S. aureus* strain ATCC BAA-41 and ampicillin-resistant *S. aureus* strain ATCC 29247, or brain heart infusion broth for antibiotic-susceptible *E. faecium* strain ATCC 49624 and vancomycin-resistant *E. faecium* strain ATCC 700221, or Mueller Hinton broth for antibiotic-susceptible strains *B. subtilis* strain 168, *E. faecalis* strain ATCC 29212 and *E. coli* strain ATCC 25922 were used in the assays. After incubation for 18 h at 37°C, the absorbance at 600 nm (A_600_) was recorded using a microplate reader (Bio-Rad laboratory Ltd., UK) and the percentage of bacterial cell inhibition with respect to vehicles (1% DMSO) was calculated. The MIC was defined as the lowest compound concentration at which the growth of bacteria was inhibited by ≥90%. Three independent assays were performed for each test.

### GTPase Activity Assay

The GTPase activity of recombinant *S. aureus* FtsZ was measured in 96-well microplates using a CytoPhos phosphate assay Biochem Kit (Cytoskeleton, USA) according to an optimized protocol and the manufacturer’s instructions. *S. aureus* FtsZ (3.5 µM) was preincubated with vehicle (1% DMSO) or different concentrations of each test compound in 50 mM 4-morpholinepropanesulfonic acid buffer (MOPS, pH 6.5) for 10 min at 25°C. Then 5 mM of MgCl_2_ and 200 mM of KCl were added. Reactions were started with the addition of 500 µM GTP and incubated at 37°C. After 30 min, the reactions were quenched by adding 100 µL of Cytophos reagent for 10 min. Inorganic phosphate was quantified by measuring the absorbance at 650 nm with a microplate reader (Bio-Rad laboratory Ltd., UK). The relative IC_50_ values were determined by nonlinear regression using a sigmoidal concentration-response curve in the Origin software v6. For the rate of GTPase activity of *S. aureus* FtsZ, different concentrations of fresh prepared FtsZ (3, 6, 9, 12, 15 µM) was preincubated with vehicle (1% DMSO) or 25 µM, 50 µM compound 2, following the same condition. The data were plotted via Origin software v6. Three independent assays were performed for all the tests.

### Light Scattering Assay

The light scattering assay was performed using a protocol adapted from the literature [39]. The polymerization and depolymerization of recombinant *S. aureus* FtsZ was measured using 90° light scattering in a thermostatically (37°C) controlled fluorescence spectrometer (Perkin Elmer, USA). Both excitation and emission wavelengths were set to 600 nm with a slit width of 2.5 nm. *S. aureus* FtsZ (6 µM) in 50 mM of MOPS buffer (pH 6.5) was incubated with vehicle (1% DMSO) or different concentrations of the test compound for 10 min at 25°C. 50 mM KCl and 10 mM MgCl_2_ were then added to establish a baseline. After 8 min, a final concentration of 1 mM GTP was added at the last fraction and the increase in light scattering was measured for an additional 2000s. The rate and extent of polymerization were measured. Appropriate blanks were subtracted from all experimental data. Moreover, in order to rule out unselective inhibition by formation of aggregates, control experiments with 0.01% (v/v) Triton X-100 from a freshly prepared 1% (v/v) stock solution were performed for berberine and compound **2** [40], [41]. Reported results are the average of three independent experiments.

### Transmission Electron Microscopy (TEM)

*S. aureus* FtsZ (12 µM) was incubated in the absence and in the presence of different concentrations of the test compounds in 50 mM MOPS buffer (pH 6.5) at 25°C. After 10 min, 5 mM MgCl_2_, 50 mM KCl, and 1 mM GTP were added to the reaction mixtures and incubated at 37°C for 15min. Then, 10 µL of the sample mixtures were placed on a glow-discharged Formvar carbon-coated copper grid (400 mesh) for 10 min. The grids were subsequently subjected to negative staining using 10 µL of 0.5% phosphotungstic acid (PTA) for 30s, air-dried and digital images of the specimen were observed with a transmission electron microscope (JEOL model JEM 2010) operated at 200 kV and equipped with a Gatan MSC 794 CCD camera.

### Bacterial Morphology and Membrane Staining Studies

The *B. subtilis* strain 168 cells were grown in LB medium. The cultures at an A_600_ of 0.01 from an overnight culture were inoculated in the same medium containing different concentrations of the test compounds and grown at 37°C for 4 h. The cells for morphology studies were harvested and resuspended in 100 µL of PBS (phosphate buffered saline) buffer containing 0.25% agarose. For membrane staining, the *B. subtilis* cells were further incubated with 1.6 µM of FM 4–64 for an additional 30 min at 37°C without shaking before harvested and resuspended in 100 µL PBS buffer containing 0.25% agarose. 10 µL of the suspension mixture were then placed on a microscopic slide pretreated with 0.1% (w/v) poly-L-lysine and the morphology of the bacterial cells was observed under a phase-contrast optical microscope at 40× magnification. The images were captured using an Olympus Bio Imaging Navigator FSX 100 microscope.

### Z-ring Visualization in *E. coli* cells

A culture of *E. coli* JM109 WM647 containing the IPTG-inducible plasmid for the overexpression of green fluorescence protein (GFP)-tagged FtsZ was grown in LB medium supplemented with 30 µg/mL of chloramphenicol. After overnight incubation, a sample of the culture was diluted to 1% in the LB medium containing different concentrations of the test compound and 40 µM of IPTG. After 4 h incubation at 37°C, the *E. coli* cells were fixed, harvested and resuspended in PBS buffer containing 0.25% of agarose. 10 µL of sample mixture were added to a pretreated microscopic slide with 0.1% (w/v) poly-L-lysine and visualized using a fluorescence microscope at 60× oil immersion magnification with a standard FITC filter set. The images were captured using an Olympus Bio Imaging Navigator FSX 100 microscope.

## Results and Discussion

### Rational Design of Berberine Derivatives Using Structure-based Drug Design

The X-ray crystal structure of *S. aureus* FtsZ was used in this study to guide the design of a new series of berberine-based inhibitors. A previous study suggested that: (1) berberine binds to a hydrophobic pocket of FtsZ which overlaps with the GTP binding site; (2) berberine cannot compete with GTP for the binding site, and (3) the berberine protons on the same flank as the quaternary ammonium of the isoquinoline core establish fewer contacts with the FtsZ enzyme than those on the opposite side [30]. As shown in Figure 1A, the GTP binding site of FtsZ is large enough to accommodate berberine without competition with the GTP substrate. However, this cavity is highly solvent exposed and predominantly polar, with the exception of a region at the top of the guanine binding site and the N-terminal end of the H7-helix. The top-scoring poses of berberine docked against the GTP binding site were found within this hydrophobic region; however, the protein-ligand shape complementarity was poor and the docking scores were relatively low. On the other hand, the C-terminal interdomain cleft is a narrow cavity delimited by a number of hydrophobic residues from the C-terminal β-sheet, the T7-loop and the H7-helix (Figure 1A). The shape and physicochemical properties of this cavity is particularly suitable for hydrophobic and planar small-molecules [24]. As shown in Figure 1B, a good fit was predicted for berberine binding into the interdomain cleft. Because the shape of the molecule is flat, berberine is particularly suitable to interact with the C-terminal four-stranded β-sheet of FtsZ. The compound establishes multiple favorable interactions with the hydrophobic side chains of Ile197, Leu200, Val203, Leu209, Met226, Leu261, Val297 and Ile311. The positively charged amine of berberine interacts with Asp199. Our predicted binding mode is also consistent with the STD NMR experimental result reported in the previous literature, suggesting that the berberine protons close to the quaternary ammonium are less engaged in FtsZ contacts [30]. Indeed, this flank of the molecule is projected towards the outside of the interdomain cavity and therefore is less accessible to interact with the protein. Visual inspection of the docking results reveals that the predicted binding pose of berberine is surprisingly similar to the experimental binding pose of PC190723 in *S. aureus* FtsZ. The molecules share a similar planarity, shape, length and alignment the ring systems. Therefore, we hypothesized that berberine binds into the C-terminal binding cleft, rather than the GTP-binding site, and the predicted binding mode was thus used for the structure-based design of new berberine derivatives.

The predicted binding mode of berberine against the interdomain cleft of FtsZ suggests that the molecule could be modified at the C9-position. The C9-methoxy substituent of berberine is pointing towards the outside of the cavity and binds into a hydrophobic region delimited by Ile228, Val230 and Val307 (Figure 1C). Because the derivatization of berberine at the C9-positon is chemically feasible [36], [42], we designed a new series of 9-phenoxyalkyl substituted berberine derivatives that are predicted to bind into the interdomain cleft of FtsZ with good scores (Figure 1C). Compounds **1–7** were synthesized and their antimicrobial and anti-FtsZ activities were tested.

### Antibacterial Activity of the 9-phenoxyalkyl Substituted Berberine Derivatives

The antibacterial activity of 9-phenoxyalkyl substituted berberine derivatives **1–7** (Figure 2) was tested against a panel of clinically relevant bacteria (Table 1). Berberine was tested under the same assay conditions as a reference compound. Consistent with the previous literature report, berberine was active against Gram-positive bacteria but less effective against Gram-negative bacteria [43]. Berberine derivatives **1–6** bearing the phenoxyalkyl group at the C9 position were not only more potent against Gram-negative bacteria, but also exhibited improved efficacy and a broader spectrum of antibacterial activity than the parent compound. Compounds **1–6** inhibited the growth of antibiotic-susceptible and antibiotic-resistant *S. aureus* strains with MIC values of 2–8 µg/mL (berberine: 128–196 µg/mL). The growth of vancomycin-susceptible and vancomycin-resistant *E. faecium* were inhibited with MIC values of 4–16 µg/mL (berberine: >196 µg/mL). Gram-negative strains *E. coli* and *K. pneumoniae* (expressing AmpC β-lactamase) were inhibited with MIC values of 32–128 µg/mL (berberine: >500 µg/mL). Compound **7** without the 9-phenoxy group was less potent than compounds **1–6**, suggesting that the aromatic ring plays an important role on the antibacterial activity. Compounds **2** and **5** with chloro- and nitro-substituents showed slightly stronger antibacterial activity than the other berberine derivatives. Most importantly, compounds **2** and **5** showed stronger antimicrobial activity than the clinically used antibiotics ampicillin and vancomycin against antibiotic-resistant bacteria *S. aureus* ATCC 29247 and ATCC BAA-41, and *E. faecium* ATCC 700221, respectively (Table 1). The same potencies of compounds **1–6** against antibiotic-sensitive and antibiotic-resistant *S. aureus* and *E. faecium* were found, implying that the berberine derivatives are not affected by the common mechanisms of bacterial antibiotic resistance. In summary, the introduction of phenoxyalkyl groups at the C9 position of berberine resulted in substantial improvements in antibacterial activity.

**Table 1:** Minimum inhibitory concentrations of 9-phenoxyalkyl substituted derivatives against a panel of bacterial strains.

Organism^a^	MIC (µg/mL)										
	1	2	3	4	5	6	7	BER	AMP	VAN	
*B. subtilis* 168	8	4	8	8	8	8	112	128	0.1	1	
*S. aureus* ATCC 29213	4	2	8	4	2	4	112	128	1.5	1	
*S. aureus* ATCC 29247	4	2	8	4	2	4	112	128	48	1	
*S. aureus* ATCC BAA-41	4	2	8	4	2	4	196	196	>96	2	
*E. faecium* ATCC 49624	8	4	16	8	8	8	>196	>196	3	1	
*E. faecium* ATCC 700221	8	4	16	8	8	8	>196	>196	6	>96	
*E. faecalis* ATCC 29212	16	4	32	8	8	16	>196	>196	6	1	
*S. epidermidis* ATCC 12228	4	2	4	4	2	4	112	128	3	1	
*E. coli* ATCC 25922	96	32	128	96	32	96	384	>500	6	2	
*K. pneumoniae* ATCC BAA-1144	128	64	128	64	64	128	>384	>500	>96	6	

Berberine (BER) was tested as a reference compound. Ampicillin (AMP) and vancomycin (VAN) were used as controls.

a *S. aureus* ATCC 29247 is ampicillin-resistant, *S. aureus* ATCC BAA-41 is methicillin-resistant and *E. faecium* ATCC 700221 is vancomycin-resistant. *K. pneumoniae* ATCC BAA-1144 is a Gram-negative strain expressing the AmpC β-lactamase.

### Inhibition of the GTPase Activity of Ftsz

**Figure 3:**
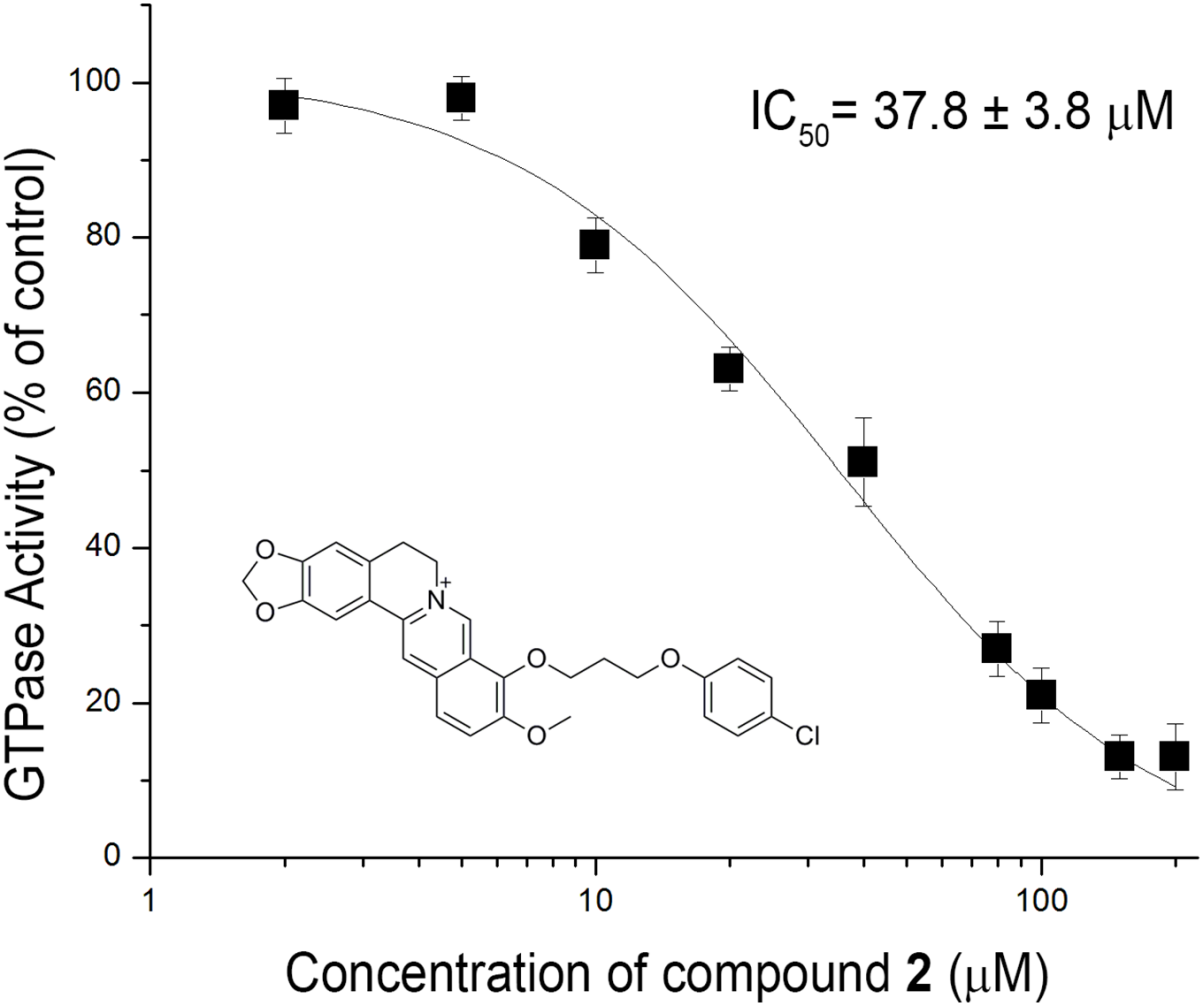
Concentration-response curve of compound 2 against the GTPase activity of *S. aureus* FtsZ. Each point represents the mean of three independent assays, and the vertical bars show the standard derivation of the mean.

Next, we tested the inhibitory effect of the 9-phenoxyalkyl substituted berberine derivatives on the GTPase activity of *S. aureus* FtsZ. Compounds **1–6** inhibited the GTPase activity with relative IC_50_ values between 37.8 and 63.7 µM. Compound **7** lacking the 9-phenoxy substituent was less potent (Table 2). The GTPase inhibition activity correlates reasonably well with the antimicrobial activity, suggesting that the compounds interfere with bacterial growth through a mechanism of FtsZ binding. Berberine was found to moderately suppress the GTPase activity of *S. aureus* FtsZ with a relative IC_50_ value of 272 µM, which is similar to a previous literature report [44]. All compounds in this study showed a typical sigmoidal decrease in the GTPase activity with increasing concentrations of the compounds. The relative concentration-response curve obtained with compound **2** against *S. aureus* FtsZ is shown in Figure 3. In the absence of compound **2**, the GTPase activity of *S. aureus* FtsZ was found to be 0.44 GTP/FtsZ/min. As shown in Figure S3, the GTPase activity of *S. aureus* FtsZ was reduced to 0.24 and 0.1 GTP/FtsZ/min in the presence of 25 µM and 50 µM of compound **2**.

**Table 2:** Effects of compounds 1–7 on *S. aureus* FtsZ GTPase activity.

Compound	IC_50_±SEM^a^ (µM)
**1**	56.8±10.4
**2**	37.8±3.8
**3**	47.3±4.6
**4**	63.7±4.8
**5**	43.4±5.2
**6**	63.5±4.7
**7**	240.43±3.3
Berberine	272±46.6

Berberine was tested as a reference molecule.

a SEM: standard error of the mean.

GTPase inhibition has also been reported with the antibacterial cell division inhibitor PC190723 [45]. It has been suggested that binding into the C-terminal interdomain cleft of FtsZ might interfere with the protein flexibility, preventing the T7-loop from exposing important catalytic residues towards the GTP binding site [26]. Taken together, the results of the GTPase inhibition assay suggest that the 9-phenoxyalkyl berberine derivatives establish stronger interactions with the FtsZ enzyme than the parent berberine, which in turn might lead to a stronger antimicrobial activity.

### Effect of the 9-phenoxyalkyl Berberine Derivatives on the Polymerization of *S. aureus* FtsZ

**Figure 4:**
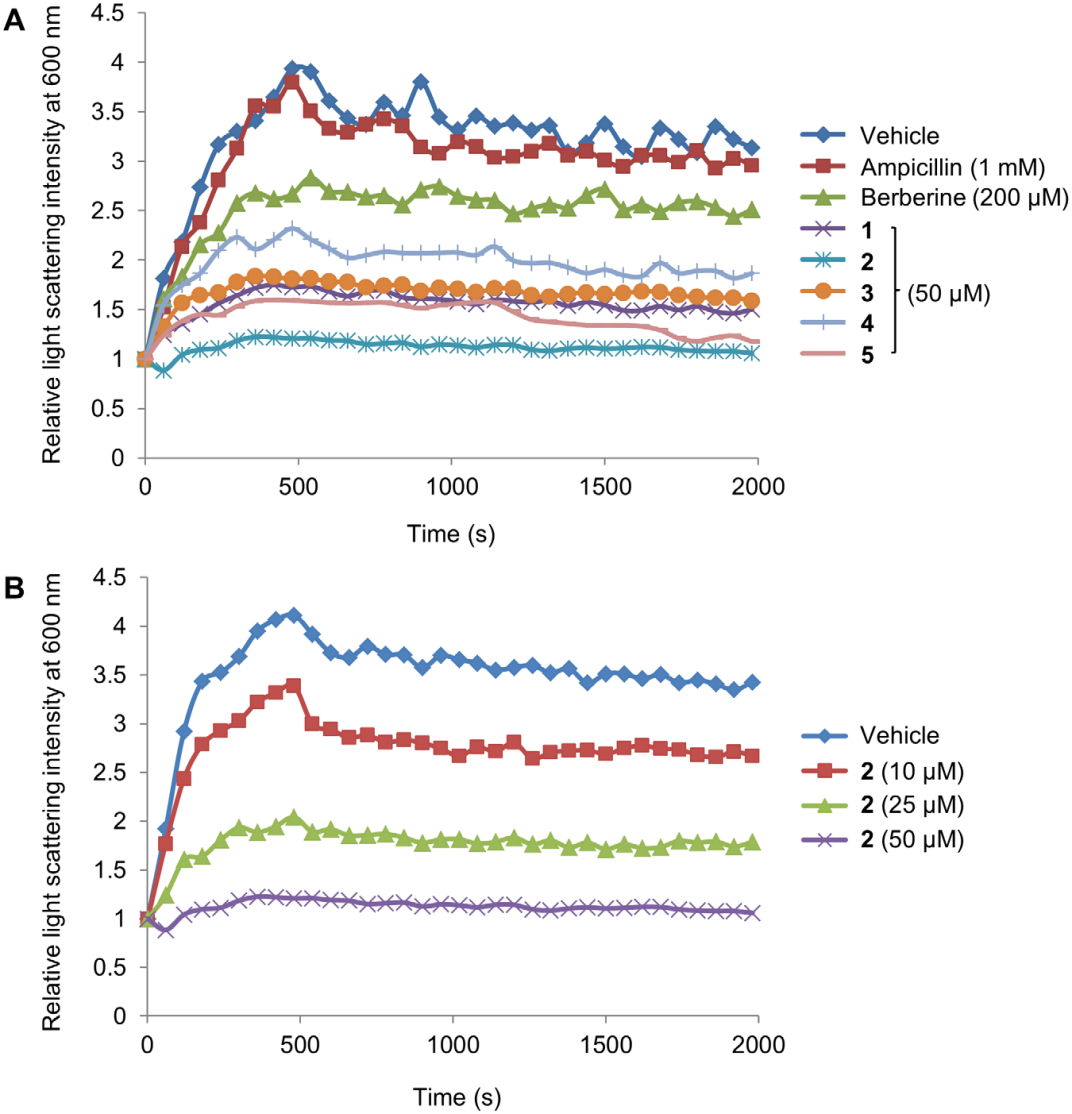
Effect of 9-phenoxyalkyl berberine derivatives on the polymerization of *S. aureus* FtsZ. (A) Effect on the polymerization of *S. aureus* FtsZ in the absence of (vehicle - 1% DMSO) or in the presence of 50 µM of compounds 1–5, 200 µM of berberine or 1 mM of ampicillin are shown. (B) The polymerizations of *S. aureus* FtsZ in the presence of compound **2** at 10 µM, 25 µM, and 50 µM are shown.

In order to gain further insight into the mechanism of antibacterial activity of 9-phenoxyalkyl substituted berberine derivatives, the effects on the self-polymerization activity of the FtsZ protein were studied. A light scattering assay was used to detect FtsZ polymerization by monitoring the increase in absorbance at 600 nm [39]. Berberine has been previously reported to inhibit the polymerization of *E. coli* FtsZ [30]. In our assay conditions berberine was able to moderately block the polymerization of *S. aureus* FtsZ, with approximately 30% inhibition at 200 µM concentration. On the other hand, compounds **1–5** at 50 µM extensively inhibited the polymerization of *S. aureus* FtsZ. The inhibition was maximal with compound **2** (∼70% inhibition, Figure 4A). The non-FtsZ-targeting antibiotic ampicillin was used in the same assay condition as a negative control. As expected, 1 mM of ampicillin had no effect on the polymerization of *S. aureus* FtsZ. Moreover, the inhibition of *S. aureus* FtsZ polymerization by compound **2** was further investigated at increasing concentrations of the molecule. As shown in Figure 4B, compound **2** strongly inhibited the *S. aureus* FtsZ polymerization in a dose-dependent manner. In order to confirm that the underlying mechanism for inhibition of FtsZ polymerization by berberine or compound **2** is not related with the formation of aggregates, similar light scattering experiments were conducted in the presence of 0.01% Triton X-100, a natural detergent that has been shown to disrupt aggregate formation in enzymatic assays [40]. As shown in Figure S1, the inhibition of polymerization by berberine and compound **2** was maintained in the presence of Triton X-100, at different concentrations of the compounds in a dose-dependent manner. These results suggest that the mechanism of inhibitory activity of berberine and its derivative **2** on FtsZ is specific rather than caused by aggregation of the compounds.

**Figure 5:**
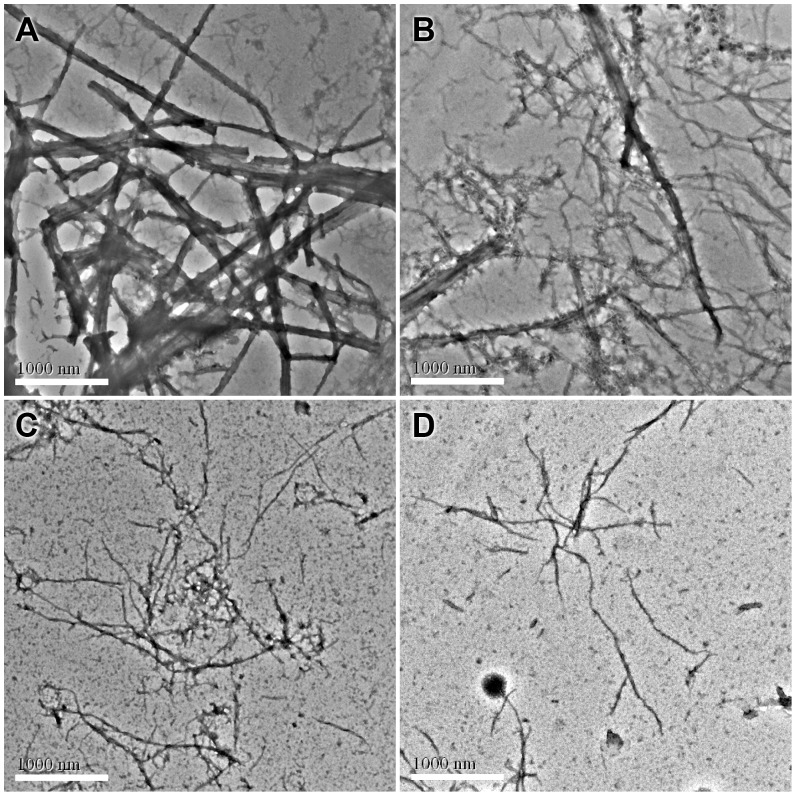
Electron micrographs of *S. aureus* FtsZ polymers in the absence and in the presence of berberine and compound 1–2. (A) vehicle (1% DMSO). (B) 200 µM of berberine. (C) 8.8****µM of compound 1. (D) 4 µM of compound 2. The length of the scale bar is 1000 nm.

The effects of 9-phenoxyalkyl substituted berberine derivatives on the polymerization of FtsZ were also analyzed by transmission electron microscopy. In the absence of the test compounds, a dense network of FtsZ protofilaments with an average width of 120±24 nm was observed (Figure 5A). Compounds **1** and **2** were found to drastically reduce the size and thickness of the FtsZ polymers and the bundling of FtsZ protofilaments at much lower concentrations than their parent compound berberine. Compounds **1** (8.8 µM) and **2** (4 µM) reduced the thickness of the bundles of FtsZ protofilaments by 60% to 70% respectively (Figures 5C and 5D). Only a few thin and short FtsZ filaments were observed in the presence of **2**.

### Effects on the Morphology and Membrane Structure of *B. subtilis* Cells

**Figure 6:**
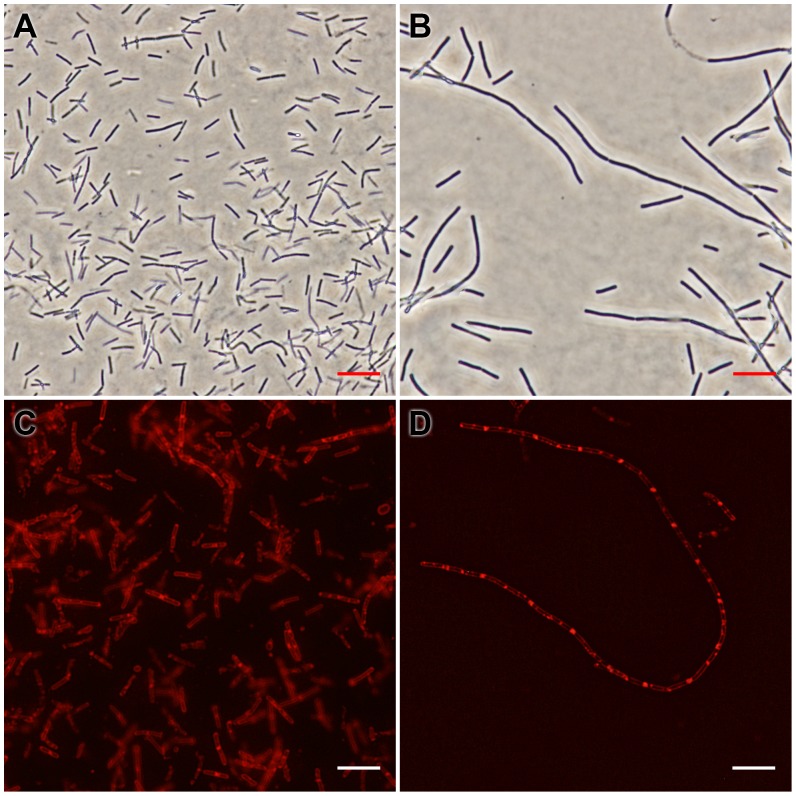
Effects of compound 2 on the cell morphology and membrane structure of *B. subtilis*. *B. subtilis* strain 168 cells were grown in the absence (A) or in the presence of 4 µM of compound **2** (B). Observations after membrane staining with the red fluorescent dye FM 4−64 are shown in the absence (C) or in the presence of 4 µM of compound 2 (D). The length of the scale bar is 10 µm.

The underlying mechanism of the antibacterial activity of compound **2** was further explored by microscopic observation of the bacterial cell morphology. Compound **2** significantly increased the cell length of *B. subtilis* (Figure. 6B), as compared to the untreated cells (Figure 6A), suggesting a mechanism of antibacterial-induced cell filamentation. It is interesting to note that similar results were also found with FtsZ inhibitors from different chemotypes, such as OBTA and PC190723 [17], [46]. This result strongly suggests that 9-phenoxyalkyl substituted berberine derivatives interact *in vivo* with the bacterial FtsZ protein. Because perturbation of membrane structure can also lead to bacterial cell lysis and death, the effect of compound **2** on the bacterial cell membrane was further investigated using a red fluorescent dye FM4−64. In spite of an increased length of *B. subtilis* cells (Figure 6D), compound **2** did not induce any detectable perturbation of the cell membrane, as compared to untreated cells (Figure 6C). These results confirm that compound **2** inhibits bacterial proliferation by inducing cell filamentation in *B. subtilis* without perturbing the bacterial membrane.

### Effects on *E. coli* Z-ring Formation

**Figure 7:**
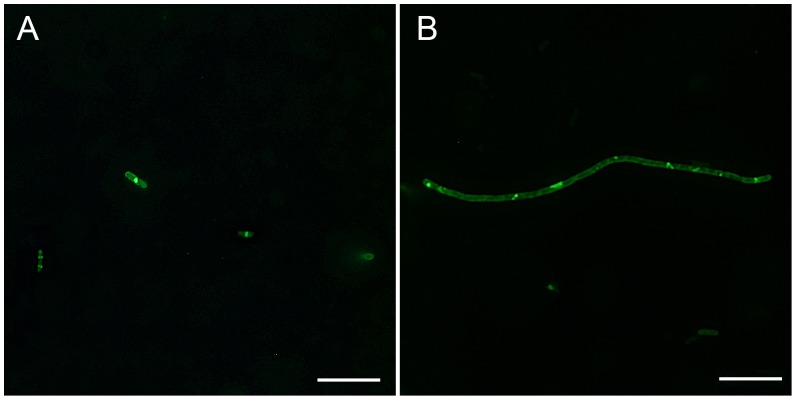
Effects of compound 2 on the Z-ring formation of *E. coli*. Perturbation of cytokinetic Z-ring formation in *E. coli* cells was visualized using green fluorescent GFP-tagged FtsZ. The bacterial cells were grown in the absence (A) or in the presence of 48 µM of compound **2** (B). The length of the scale bar is 10 µm.

To confirm that 9-phenoxyalkyl substituted berberine derivatives target at the FtsZ protein *in vivo*, the formation of the dynamic Z-ring in the Gram-negative *E. coli* cells was studied. During bacterial cell division, rod-shaped *E. coli* initializes a septum formation by the dynamic Z-ring. Previous studies have shown that perturbation of the FtsZ function could lead to the inhibition of bacterial proliferation by a mechanism of interference with the Z-ring formation [14], [30], [46]. In the absence of compound **2**, a green fluorescent band of GFP-tagged FtsZ was visible at the cell midpoint (Figure 7A). The fluorescent bands represent septation-competent localized Z-rings (cytoskeletal structures). In the presence of 48 µM of compound **2**, GFP-tagged FtsZ dispersed as discrete foci throughout the elongated cells (Figures 7B and Figure S2), indicating that compound **2** caused mislocalization of the FtsZ protein.

Taken together, the *in vivo* and *in vitro* results confirmed the role of 9-phenoxyalkyl substituted berberine derivatives as FtsZ inhibitors, with much stronger potency than the parent compound berberine.

## Conclusion

Natural products and semi-synthetic derivatives provide a rich source of bioactive compounds for the development of new antibacterial agents. However, in the past decade most of the new chemical entities that reached the clinical practice were derived from the same natural scaffolds [47]. Berberine has been traditionally used to treat microbial infections. At the doses commonly used, the compound is considered safe [27], [48]. Recent studies confirmed that berberine is a moderate inhibitor of FtsZ, an important bacterial cell division protein. In this study, molecular docking simulations suggested that berberine binds into the C-terminal interdomain cleft of FtsZ, projecting the 9-methoxy group towards the outside of the cavity. Based on the docking results, a new series of 9-phenoxyalkyl berberine derivatives was hypothesized to establish additional favorable interactions with FtsZ.

The 9-phenoxyalkyl substituted derivatives exhibited potent antimicrobial activity against Gram-positive bacterial strains such as ampicillin- and methicillin-resistant *S. aureus*, and broader spectrum of activity than the parent compound berberine. Biochemical evaluations demonstrated that the new berberine derivatives target the bacterial FtsZ protein. The compounds were potent inhibitors of the GTPase activity of FtsZ and were able to inhibit the FtsZ polymerization in a dose-dependent manner. These results suggest that the binding of berberine derivatives into the interdomain cleft interferes with the GTPase activity of FtsZ, which in turn destabilizes the formation of FtsZ polymers. In summary, the results of this study demonstrate the potential of the berberine scaffold for chemical optimization into potent inhibitors of FtsZ with broad-spectrum antibacterial activity.

## Supporting Information

Figure S1 Control experiments in the absence and in the presence of 0.01% Triton X-100 to study the effect of berberine and its 9-phenoxyalkyl derivative 2 on the polymerization of *S. aureus* FtsZ. (A) Effect on the polymerization of *S. aureus* FtsZ by 200 µM and 500 µM of berberine. (B) Effect on the polymerization of *S. aureus* FtsZ by 10 µM and 25 µM of compound **2**.
(TIF)

Figure S2 Effects of compound 2 on the Z-ring formation of *E. coli*. Perturbation of cytokinetic Z-ring formation in *E. coli* cells was visualized using green fluorescent GFP-tagged FtsZ. The bacterial cells were grown in the presence of 48 µM of compound **2**. The length of the scale bar is 10 µm.
(TIF)

Figure S3 Inhibition of *S. aureus* FtsZ GTPase activity by 25 µM and 50 µM of compound 2 at increasing FtsZ concentrations.
(TIF)

Text S1 Supplemental information, including synthesis of berberine derivatives, ^1^H NMR and mass spectra of berberine derivatives and references.
(PDF)
