# Supplementary figures and images for: Rational Design of Berberine-Based FtsZ Inhibitors with Broad-Spectrum Antibacterial Activity
Source: PLoS One. 2014 May 13;9(5):e97514. doi: 10.1371/journal.pone.0097514 (PMC4019636; doi:10.1371/journal.pone.0097514)

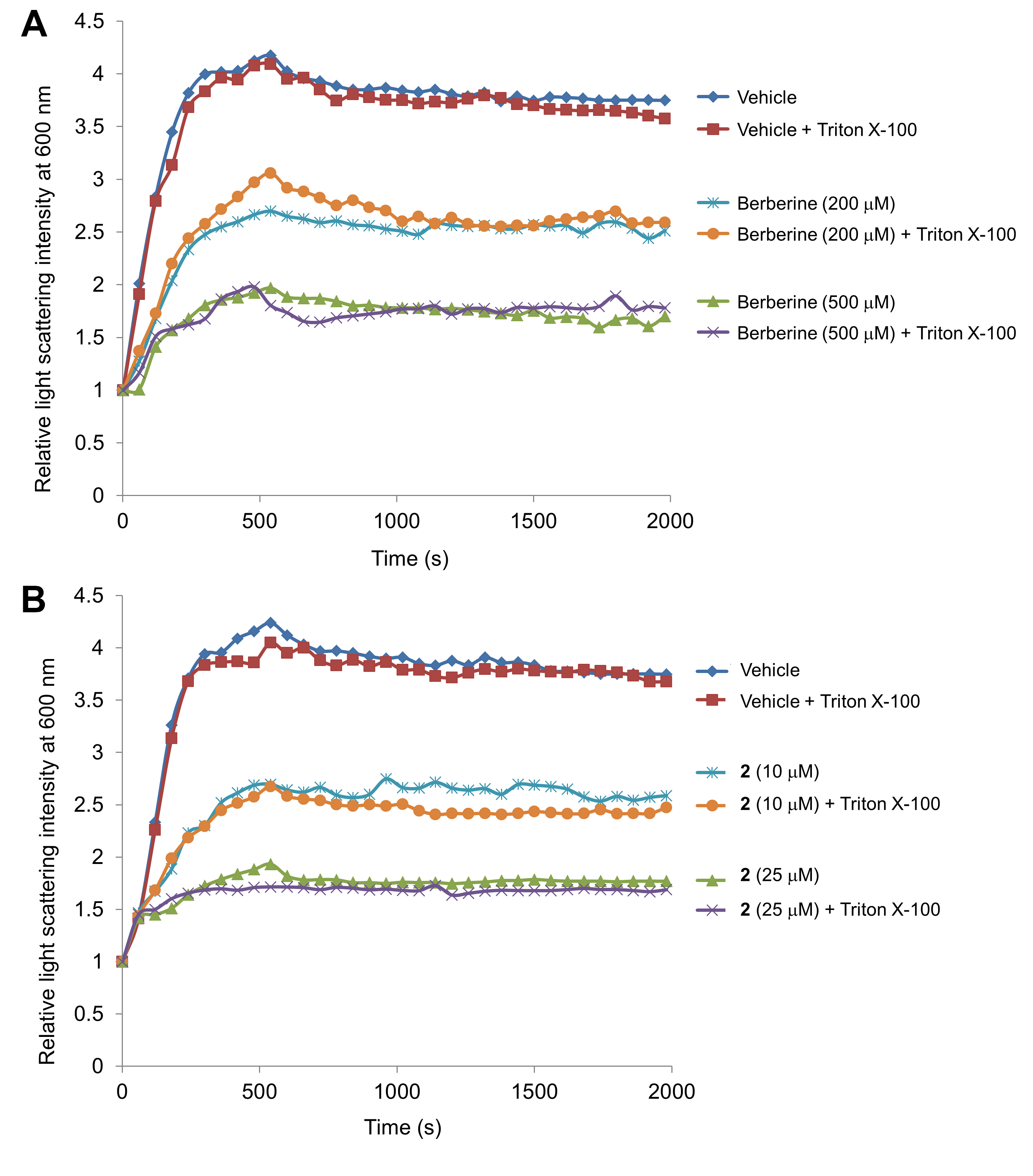

Supplement: Figure S1 — Control experiments in the absence and in the presence of 0.01% Triton X-100 to study the effect of berberine and its 9-phenoxyalkyl derivative 2 on the polymerization of S. aureus FtsZ. (A) Effect on the polymerization of S. aureus FtsZ by 200 µM and 500 µM of berberine. (B) Effect on the polymerization of S. aureus FtsZ by 10 µM and 25 µM of compound 2. (TIF) [file pone.0097514.s001.tif]

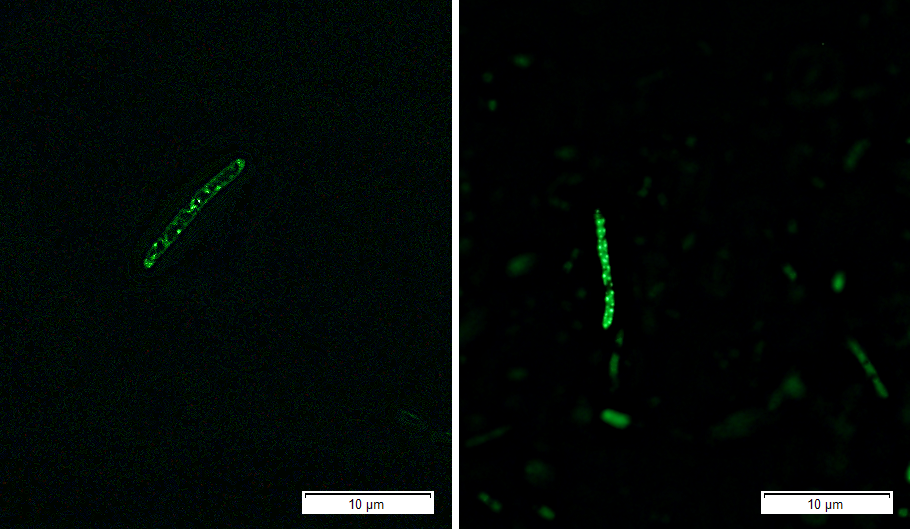

Supplement: Figure S2 — Effects of compound 2 on the Z-ring formation of E. coli. Perturbation of cytokinetic Z-ring formation in E. coli cells was visualized using green fluorescent GFP-tagged FtsZ. The bacterial cells were grown in the presence of 48 µM of compound 2. The length of the scale bar is 10 µm. (TIF) [file pone.0097514.s002.tif]
