# Supplementary material for: Rational Design of Berberine-Based FtsZ Inhibitors with Broad-Spectrum Antibacterial Activity
Source: PLoS One. 2014 May 13;9(5):e97514. doi: 10.1371/journal.pone.0097514 (PMC4019636; doi:10.1371/journal.pone.0097514)
Supplement: Text S1 — Supplemental information, including synthesis of berberine derivatives, 1H NMR and mass spectra of berberine derivatives and references. (PDF) [file pone.0097514.s004.pdf]

Text S1 of Supporting Information

Rational Design of Berberine-Based FtsZ Inhibitors with Broad-Spectrum Antibacterial Activity

Ning Sun[1], Fung-Yi Chan[1,3], Yu-Jing Lu[1,2], Marco A. C. Neves[3], Hok-Kiu Lui[1], Yong Wang[1], Ka-Yan Chow[1], Kin-Fai Chan[1], Siu-Cheong Yan[1], Yun-Chung Leung[1], Ruben Abagyan[3,4], Tak-Hang Chan[1], Kwok-Yin Wong[1*]

[1] Department of Applied Biology and Chemical Technology and the State Key Laboratory of Chirosciences, The Hong Kong Polytechnic University, Hunghom, Kowloon, Hong Kong, China.

[2] School of Chemical Engineering and Light Industry, Guangdong University of Technology, Guangzhou 510006, China.

[3] Skaggs School of Pharmacy & Pharmaceutical Sciences, University of California, San Diego, 9500 Gilman Drive, La Jolla, CA 92093, USA.

[4] Molsoft L.L.C, 11199 Sorrento Valley Road, S209, San Diego, CA 92121, USA.

[*] Address correspondence to: Professor Kwok-yin Wong, Department of Applied Biology and Chemical Technology and the State Key Laboratory of Chiosciences, The Hong Kong Polytechnic University, Hung Hom, Kowloon, Hong Kong, P. R. China. Tel: (852) 3400 3977, Fax: (852) 2364 9932, Email: kwok-yin.wong@polyu.edu.hk

Contents:

Page S2-S4: Synthesis of berberine derivatives
Page S5-S12: ESI-Mass spetra of berberrubine and compounds **1-7**
Page S13-S20: ^{1}H NMR spetra of berberrubine and compounds **1-7**
Page S21: References

Synthesis of berberine derivatives

The berberine derivbatives were prepared according to previous protocols [1]. The synthetic pathway was shown in Scheme1. Firstly, the ω-bromoalkyl ether derivatives were synthesized from the commercially available phenol derivatives, which reacted with α,ω-dibromoalkanes in the presence of potassium carbonate in butanone to give a good yield [1]. Then, the selective demethylation of berberine at 190 ℃ under the vacuum gave a 68% yield of berberrubine [2]. Finally, the target compounds were obtained by reaction of berberrubine with different ω-bromoalkyl ether derivatives in acetonitrile for 12-24 h respectively [1].

Preparation of berberrubine

To remove the methyl group at the C9-position of berberine, 5 mmol berberine was heated at 190ºC under vacuum for 30-50 min until the powder turned into dark brown. The powder was then purified by flash chromatography (CH_2Cl_2 / MeOH = 10:1) to yield a carmine powder after solvent removal. Yield: 68%. ^{1}H NMR (400 MHz, DMSO-*d6*): δ 3.04 (t, *J* = 6.0 Hz, 2H), 3.73 (s, 3H), 4.8 (t, *J* = 6.0 Hz, 2H), 6.1 (s, 2H), 6.36 (d, *J* = 8.0 Hz, 1H), 6.96 (s, 1H), 7.16 (d, *J* = 7.6 Hz, 1H), 7.61 (s, 1H), 7.98 (s, 1H), 9.07 (s, 1H). ESI-MS m/z: 322.1 $[M + H]^+$.

Preparation of compounds **1**-**7**

Berberrubine (0.15 mmol) and 1,3-dibromopropane (0.5 mmol), for the synthesis of compound **7**, or a ω-bromoalkyl ether derivative (0.5 mmol) for synthesis of **1**-**6** were mixed in 10 mL acetonitrile and stirred for 12 h at 90ºC. After reaction, the crude product was obtained by removal of organic solvent under vacuum and then purified by flash chromatography (CH_2Cl_2 / MeOH = 20:1) to yield a yellow powder. Yield: 40-55 %. ^{1}H NMR (400 MHz, DMSO-d_6):

9-O-[3(Phenylol-1-yloxyl)propyl]-berberine bromide (**1**): δ 3.35 (t, *J* = 6.4 Hz, 2H), 3.22 (t, *J* = 6.0 Hz, 2H), 4.01(s, 3H), 4.27 (t, *J* = 6.4 Hz, 2H), 4.49 (t, *J* = 6.4 Hz, 2H), 4.87 (t, *J* = 6.0 Hz, 2H), 6.18 (s, 1H), 6.93-6.99 (m, 3H), 7.10 (s, 1H), 7.31 (t, *J* = 7.6 Hz, 2H), 7.80 (s, 1H), 8.05 (d, *J* = 8.8 Hz, 1H), 8.20 (d, *J* = 9.2 Hz, 1H), 8.94 (s, 1H), 9.78 (s, 1H); ESI-MS m/z: 456.2 $[M - Br]^+$

9-O-[3-(4-Chloro-phenoxyl)propyl]-berberine bromide (**2**): δ 2.31-2.37 (m, 2H), 3.2 (t, *J* = 6.4 Hz, 2H). 4.01 (s, 3H), 4.26 (t, *J* = 6.4 Hz, 2H), 4.70 (t, *J* = 6.4 Hz, 2H), 4.90 (t, *J* = 6.4 Hz, 2H), 6.18 (s, 2H), 7.01 (d, *J* = 6.0 Hz, 1H), 7.10 (s, 1H), 7.35 (d, *J* = 6.0 Hz, 1H), 7.81 (s, 1H), 7.99 (d, *J* = 5.2 Hz, 1H), 8.19 (d, *J* = 5.2 Hz, 1H), 8.94 (s, 1H), 9.79 (s, 1H); ESI-MS m/z: 490.1 $[M - Br]^+$

9-O-[3-(4-Methoxy-phenoxyl)propyl]-berberine bromide (**3**): δ 2.29 (t, *J* = 6.4 Hz, 2H), 3.17 (t, *J* = 6.0 Hz, 2H), 3.67 (s, 3H), 4.00 (s, 3H), 4.17 (t, *J* = 6.4 Hz, 2H), 4.45 (t, *J* = 6.4 Hz, 2H), 4.85 (t, *J* = 6.0 Hz, 2H), 6.16 (s, 2H), 6.83-6.89 (m, 4H), 7.07 (s, 1H), 7,78 (s,1H), 7.97 (d, *J* = 8.8 Hz, 1H), 8.17 (t, *J* = 8.8 Hz, 1H), 8.91 (s, 1H), 9.75 (s, 1H); ESI-MS m/z: 486.2 $[M - Br]^+$

9-O-[3-(2-Methyl-phenoxyl)propyl]--berberine bromide (**4**): δ 2.14 (s, 3H), 2.36 (m, 2H), 3.18 (t, *J* = 6.0 Hz, 2H), 3.99 (s, 3H), 4.27 (t, *J* = 6.0 Hz, 2H), 4.52 (t, *J* = 6.4 Hz, 2H), 4.86 (t, *J* = 6.4 Hz, 2H), 6.18 (s, 2H), 6.85 (t, *J* = 8.0 Hz, 1H), 7.00 (d, *J* = 8.0 Hz, 1H), 7.09 (s, 1H), 7.13-7.19 (m, 2H), 7.81 (s, 1H), 8.10 (d, *J* = 9.2 Hz, 1H), 8.19 (d, *J* = 9.2 Hz, 1H), 8.96 (s, 1H), 9.78 (s, 1H); ESI-MS m/z: 470.2 $[M - Br]^+$

9-O-[3-(4-Nitro-phenoxyl)propyl]-berberine bromide (**5**): δ 2.36-2.42 (m, 2H), 3.20 (t, *J* = 6.0 Hz, 2H), 4.00 (s, 3H), 4.43 (t, *J* = 6.4 Hz, 2H), 4.48 (t, *J* = 6.4 Hz, 2H), 4.92 (t, *J* = 6.0 Hz, 2H), 6.18 (s, 2H), 7.10 (s, 1H), 7.20 (d, *J* = 2.0 Hz, 2H), 7.80 (s, 1H), 8.00 (d, *J* = 7.2 Hz, 1H), 8.10 (d, *J* = 7.2 Hz, 1H), 8.24 (d, *J* = 2.0 Hz, 2H), 8.93 (s,1H), 9.81 (s, 1H); ESI-MS m/z: 501.2 $[M - Br]^+$

9-O-[4(Phenylol-1-yloxyl)butyl]-berberine bromide (**6**): δ 2.01-2.09 (m, 4H), 3.20 (t, *J* = 6.4 Hz, 2H), 4.05 (s, 3H), 4.09 (t, *J* = 6.0 Hz, 2H), 3.37 (t, *J* = 6.0 Hz, 2H), 4.94 (t, *J* = 6.4 Hz, 2H), 6.18 (s, 2H), 6.91-6.95 (m, 3H), 7.10 (s, 1H), 7.29 (t, *J* = 7.2 Hz, 2H), 7.81 (s,1H), 7.99 (d, *J* = 9.2 Hz, 1H), 8.21 (d, *J* = 9.2 Hz, 1H), 8.94 (s, 1H), 9.78 (s, 1H); ESI-MS m/z: 470.2 $[M - Br]^+$

9-O-(3-Bromopropoxy)-berberine bromide (**7**): δ 2.40-2.46 (m, 2H), 3.22 (t, J = 6.0 Hz, 2H), 3.83 (t, J = 6.4 Hz, 2H), 4.08 (s, 3H), 4.21 (t, J = 6.4 Hz, 2H), 4.95 (t, J = 6.0 Hz, 2H), 6.19 (s, 2H), 7.10 (s, 1H), 7.81 (s, 1H), 8.10 (d, J = 9.2 Hz, 1H), 8.21 (d, J = 9.2 Hz, 1H), 8.95 (s, 1H), 9.81 (s, 1H); ESI-MS m/z: 442.1 [M - Br]+

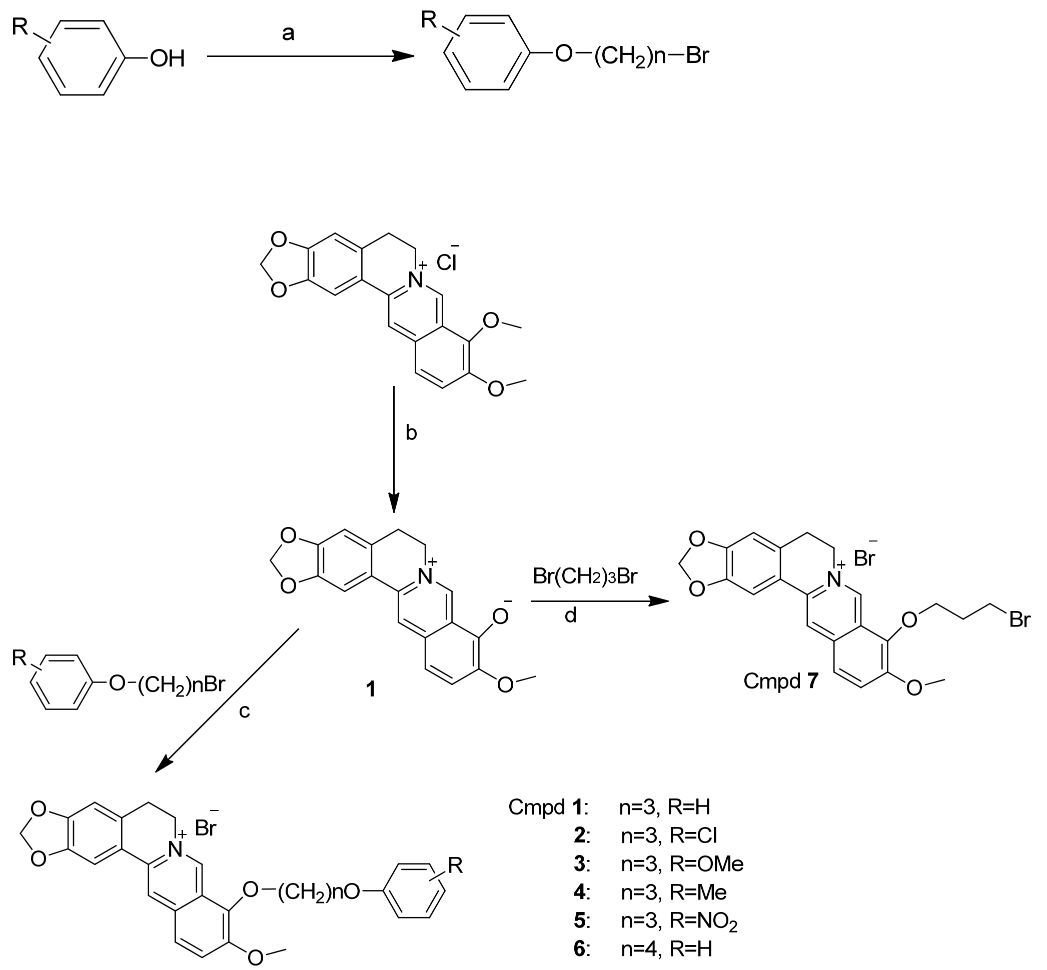

Scheme 1 Synthetic routes of berberine derivatives.

Reagents and conditions: (a) $Br(CH_2)nBr$ (n=3 or 4), K_2CO_3, Butanone, 70 ℃, 2 h; (b) 190 ℃, under vacuum, 30 min; (c) (d) CH_3CN, 80 ℃.

Mass spectra of compounds

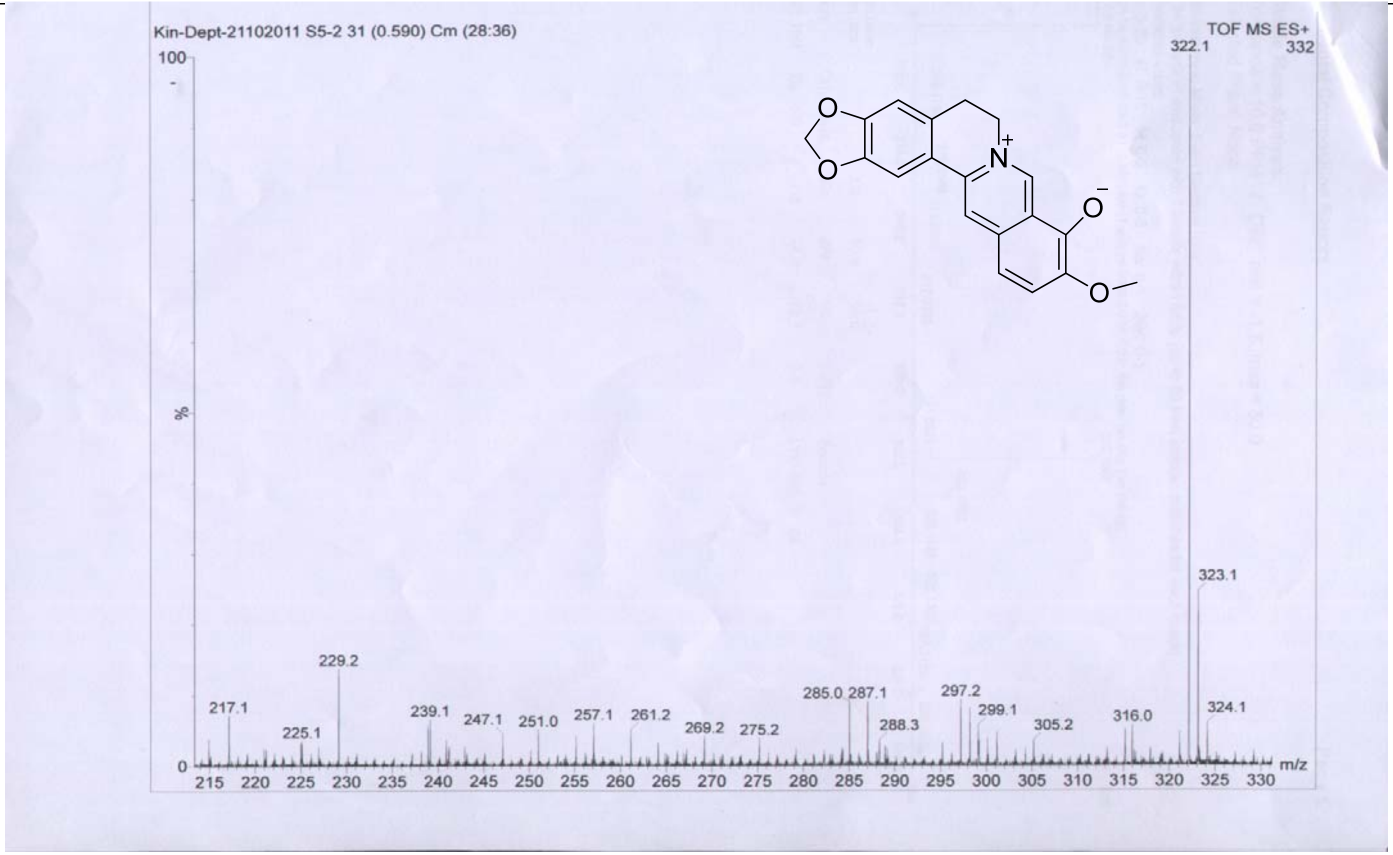

Mass spectrum of berberrubine

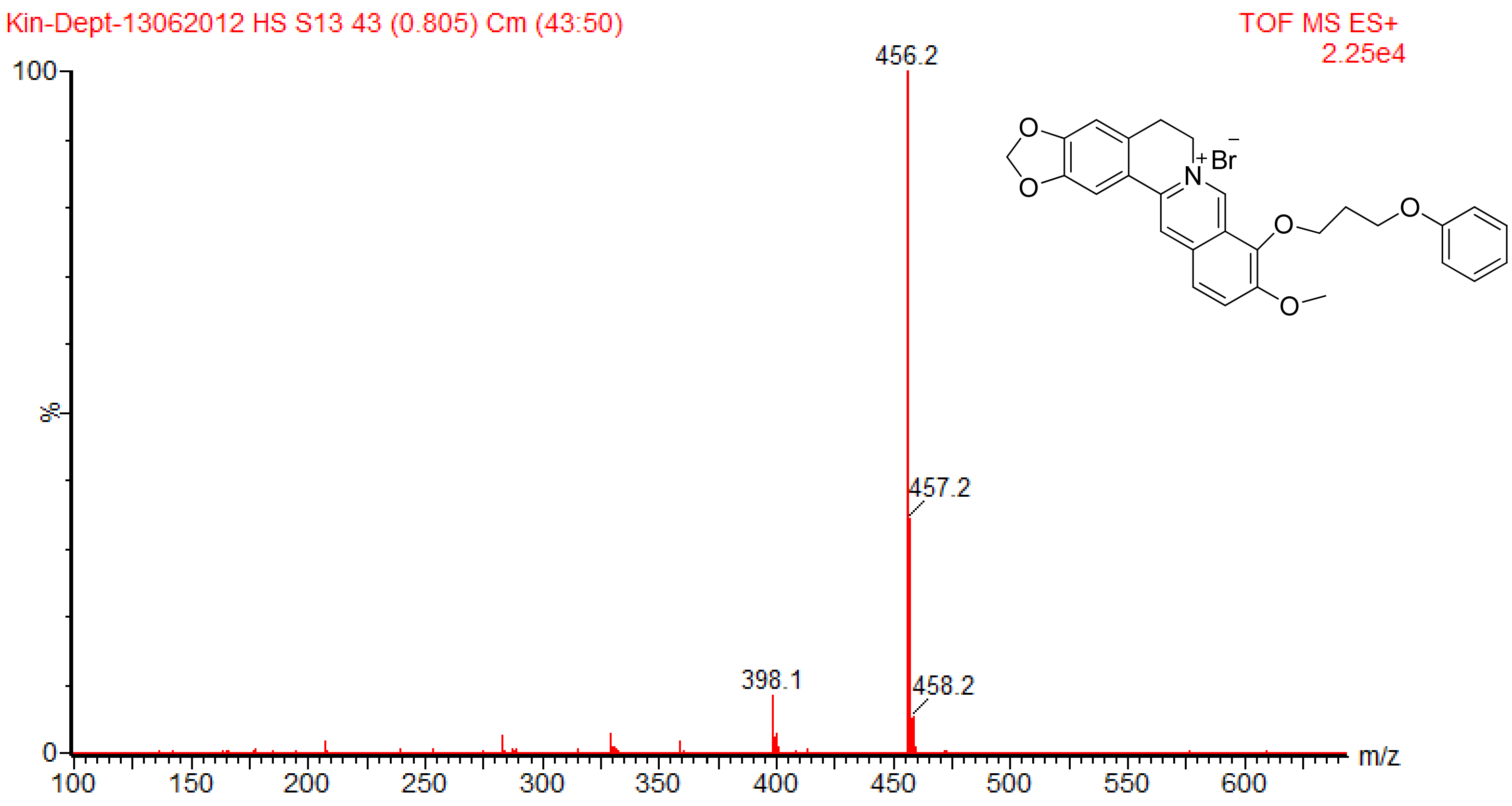

Mass spectrum of compound **1**

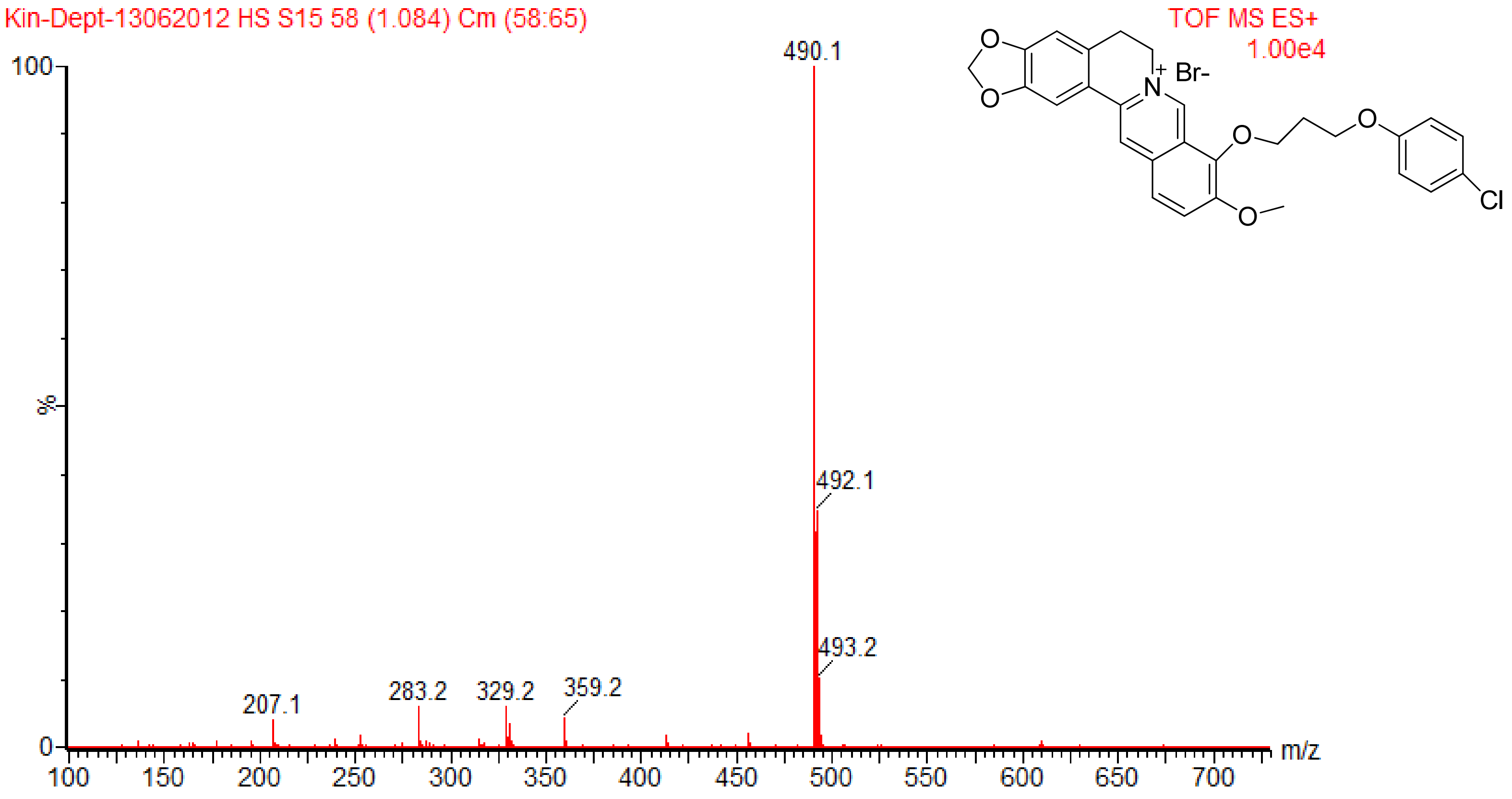

Mass spectrum of compound **2**

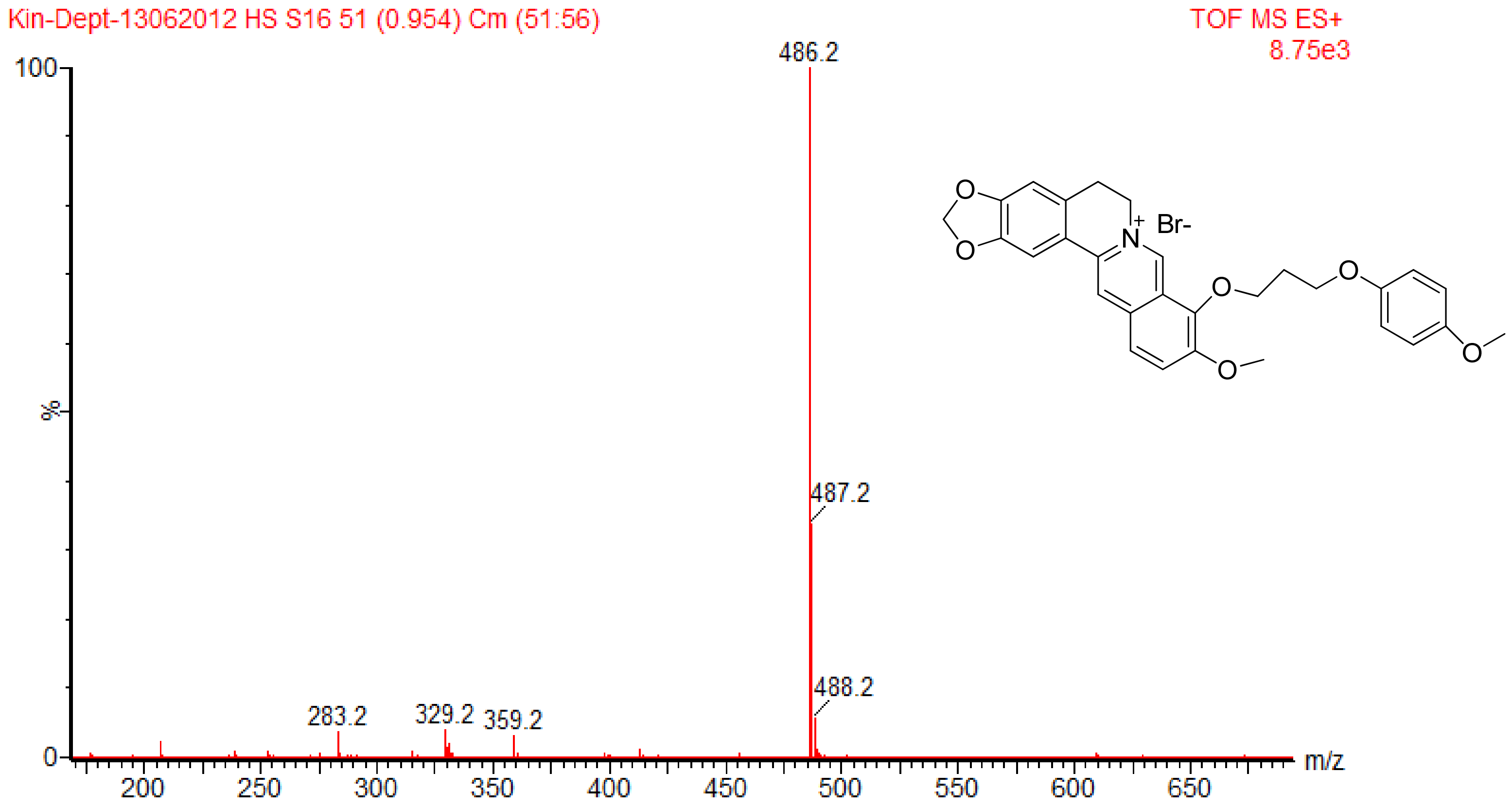

Mass spectrum of compound **3**

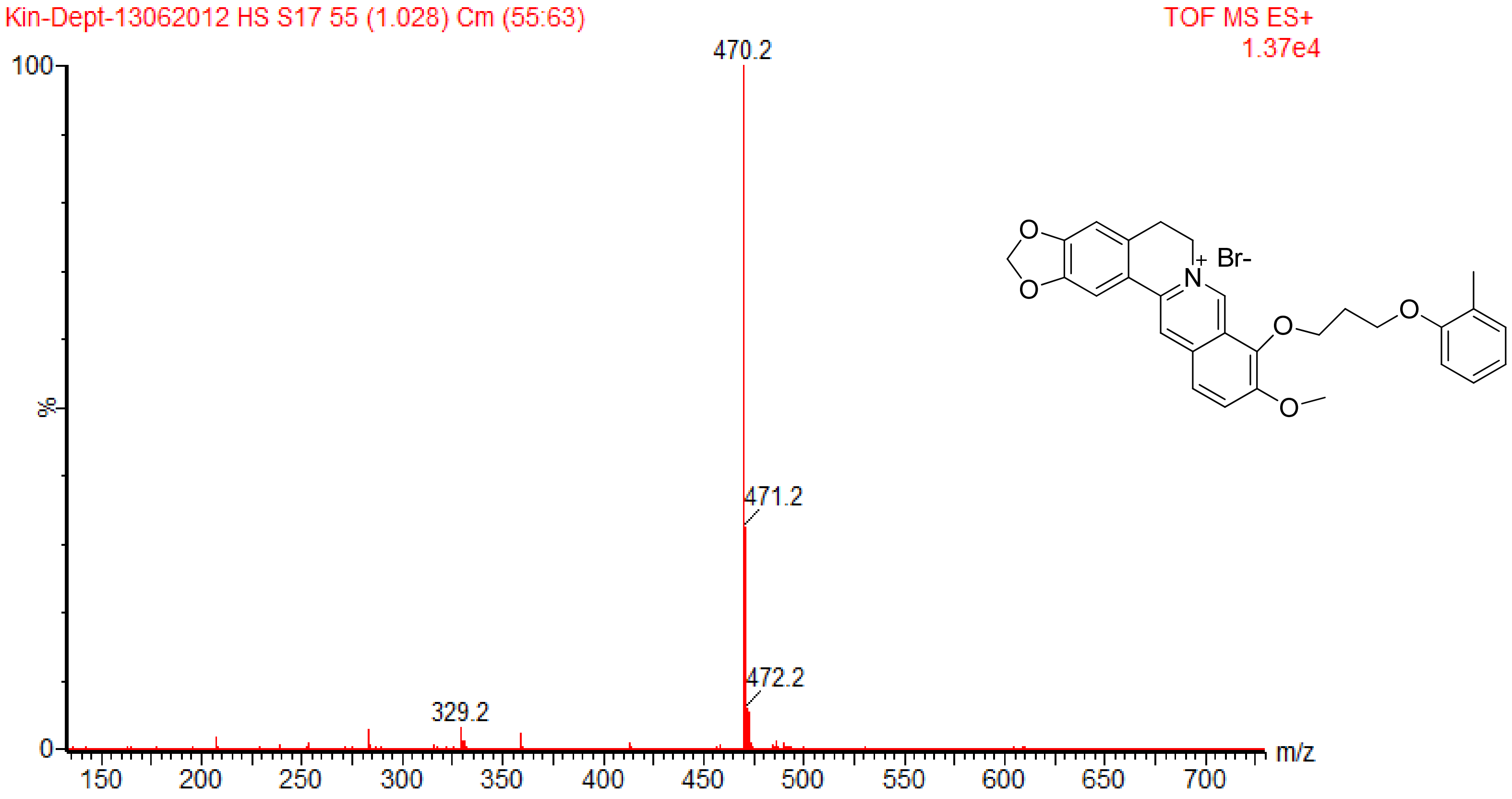

Mass spectrum of compound **4**

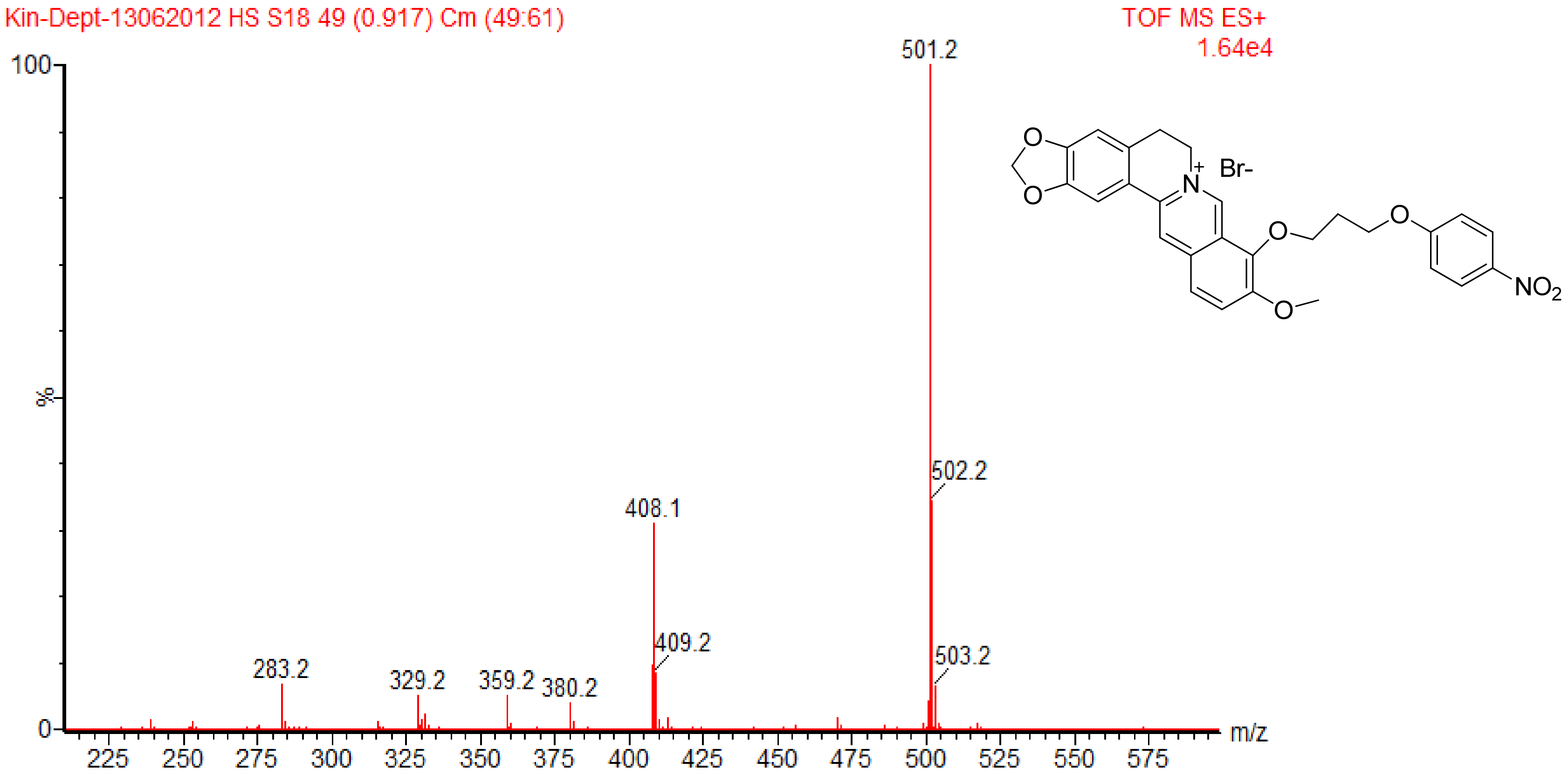

Mass spectrum of compound **5**

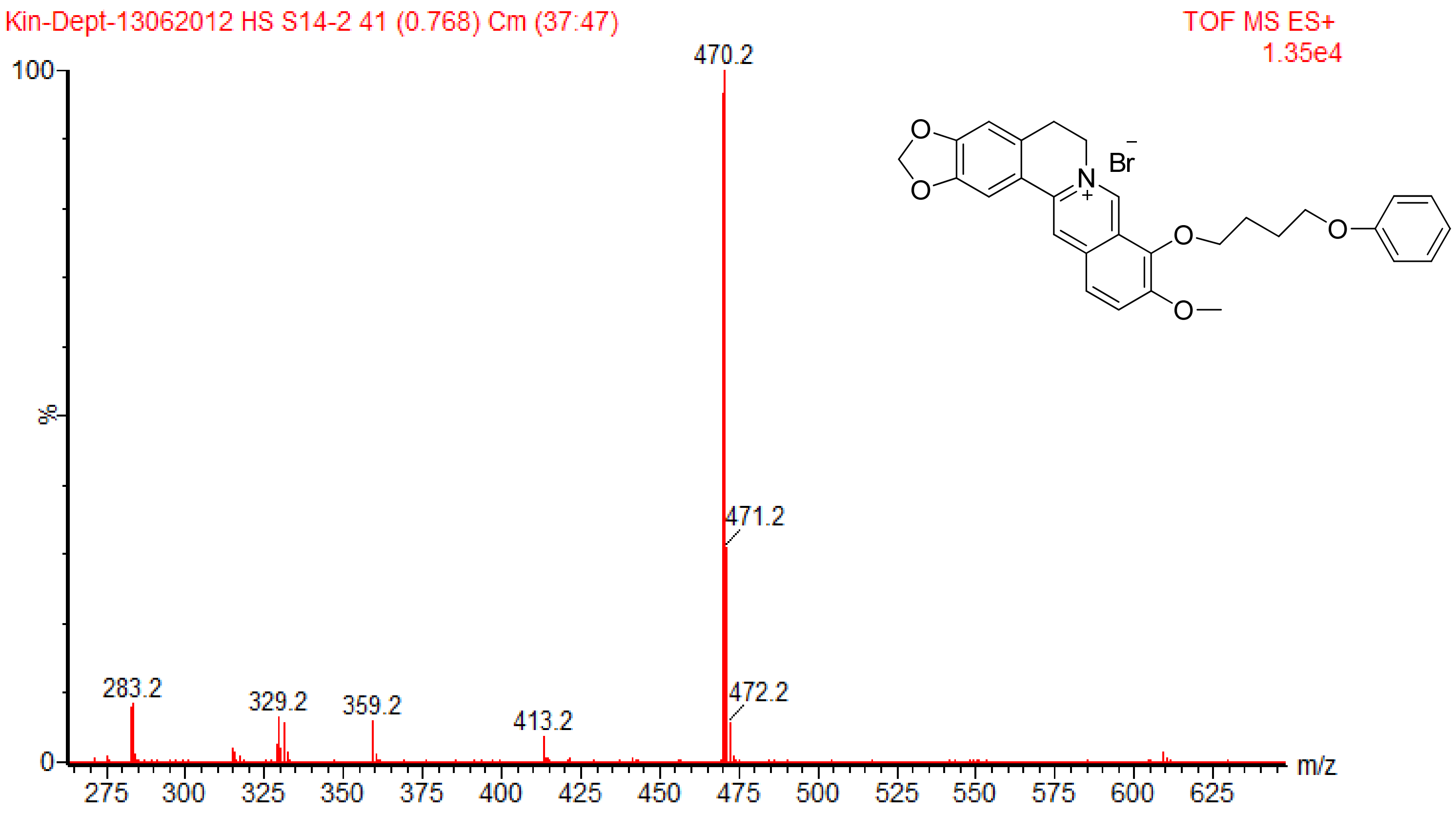

Mass spectrum of compound **6**

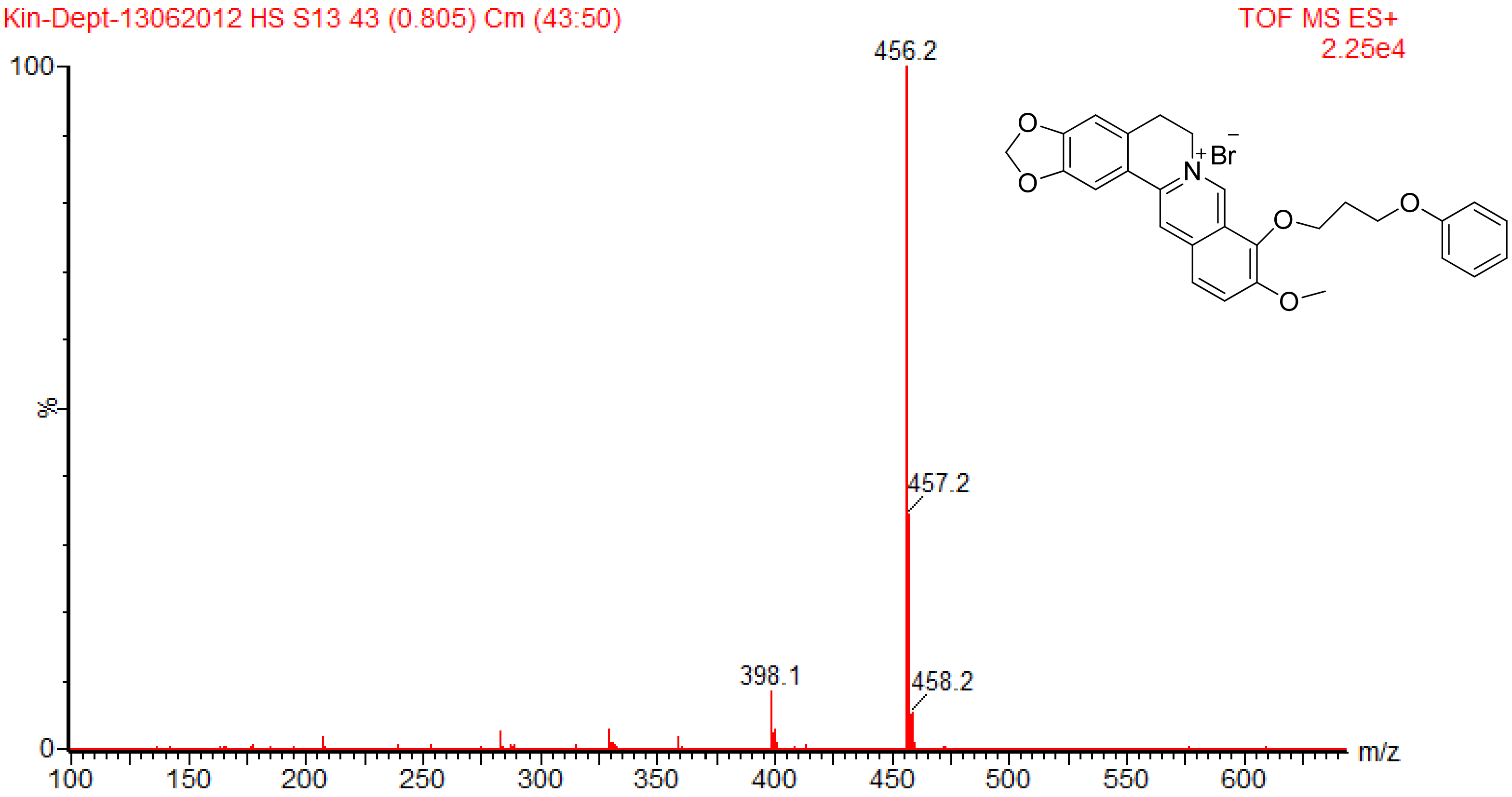

Mass spectrum of compound **7**

^{1}H NMR spectra of compounds

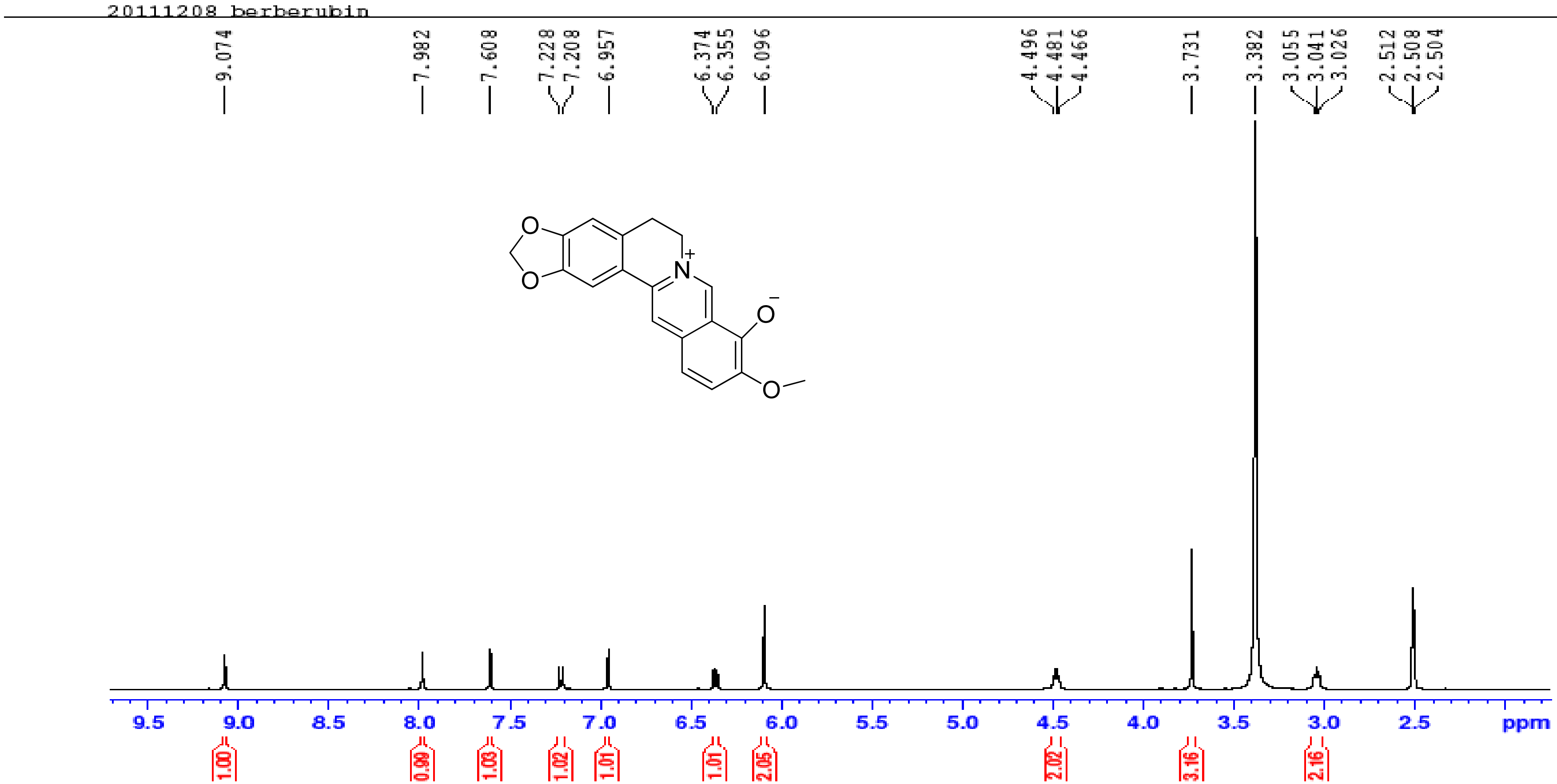

^{1}H NMR spectrum of berberrubine

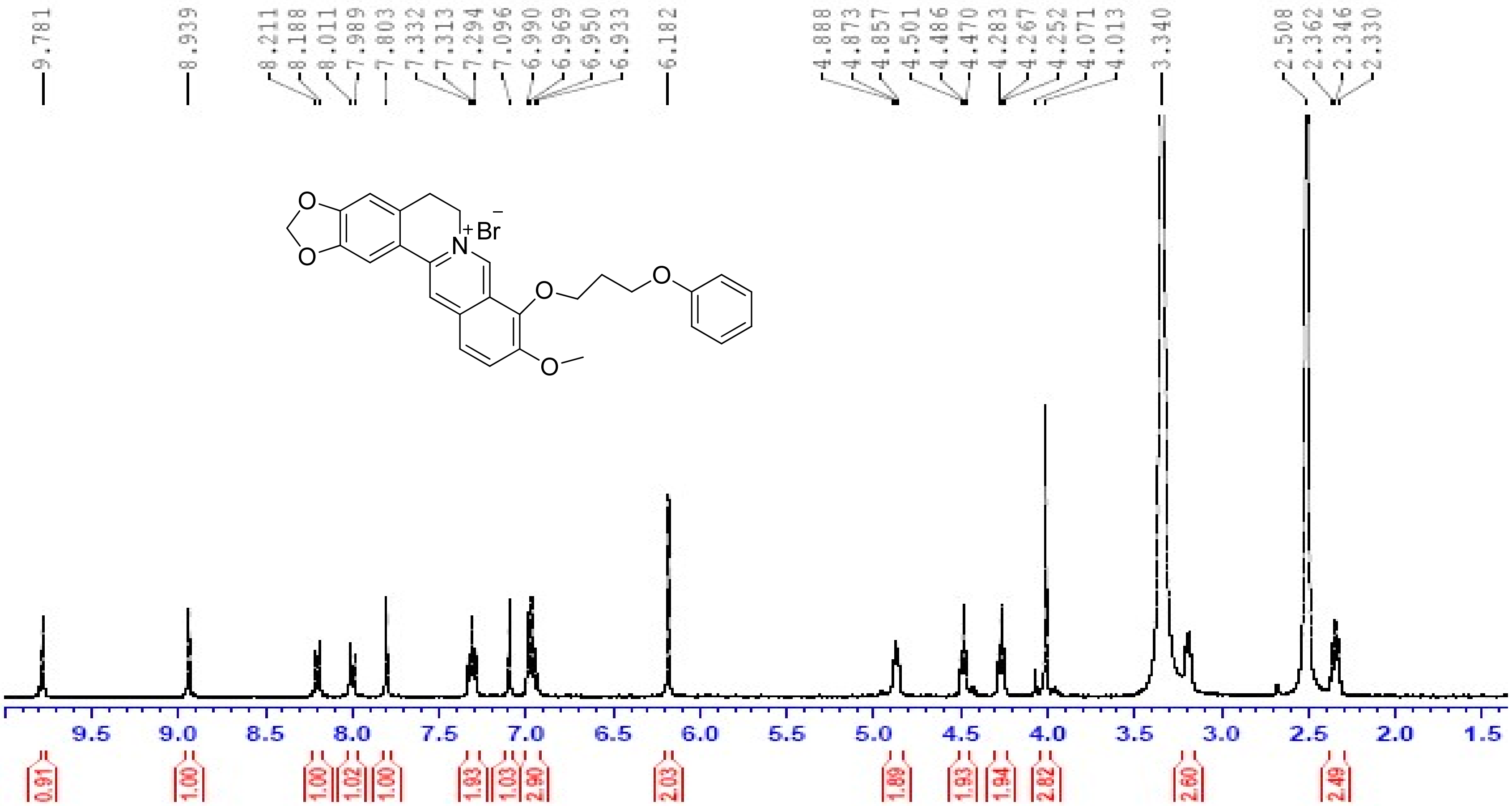

^{1}H NMR spectrum of compound **1**

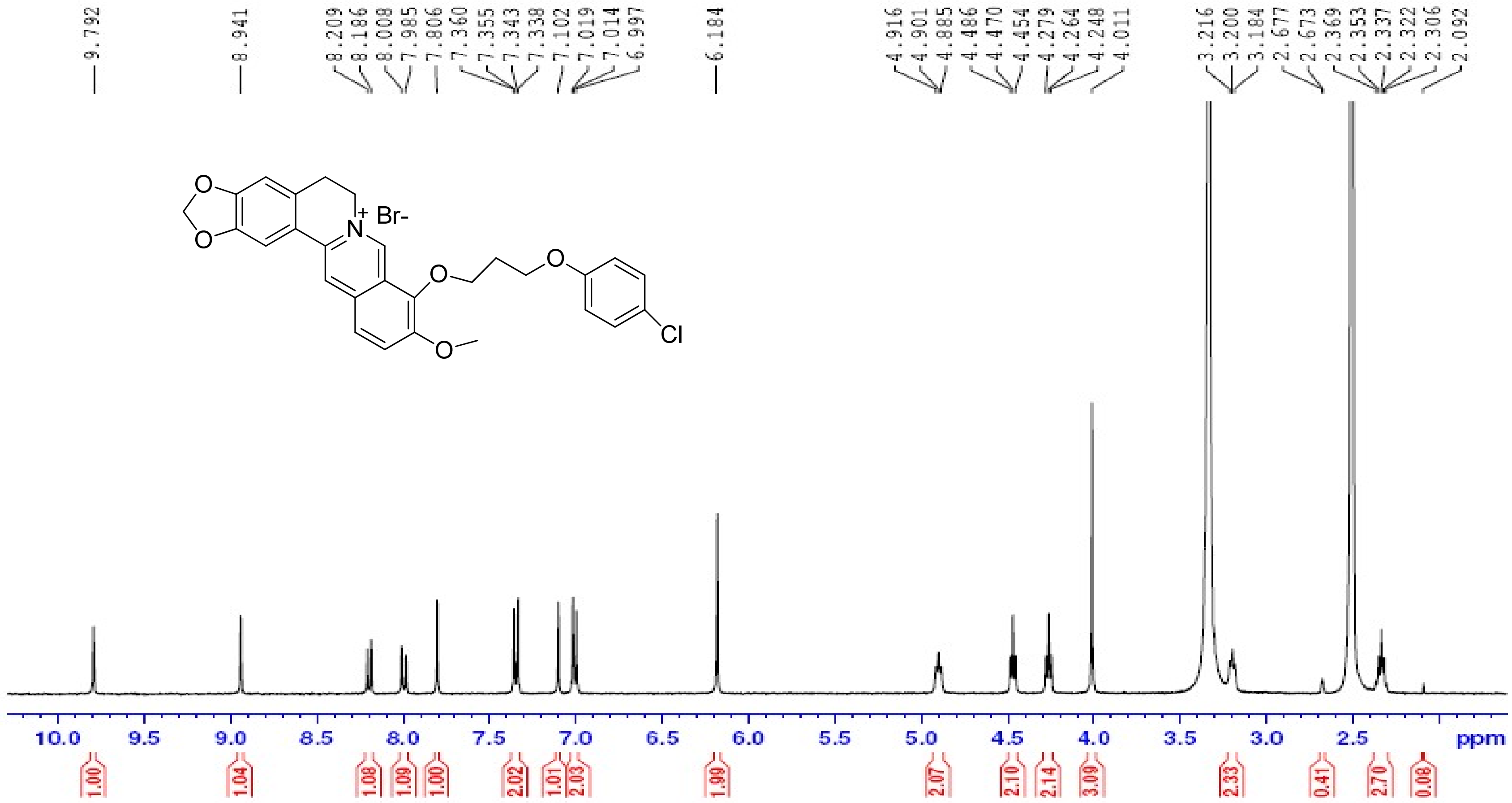

^{1}H NMR spectrum of compound **2**

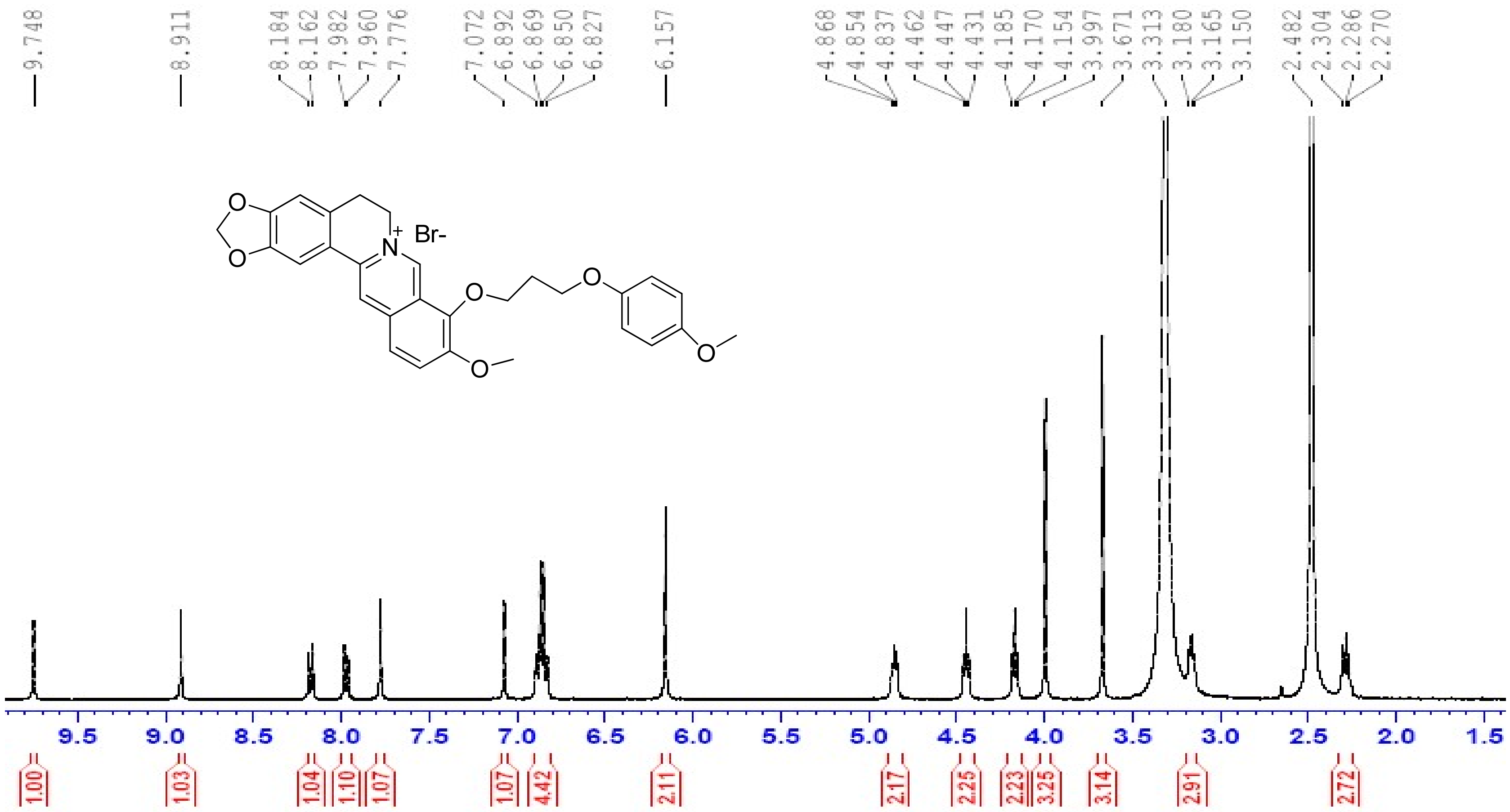

^{1}H NMR spectrum of compound **3**

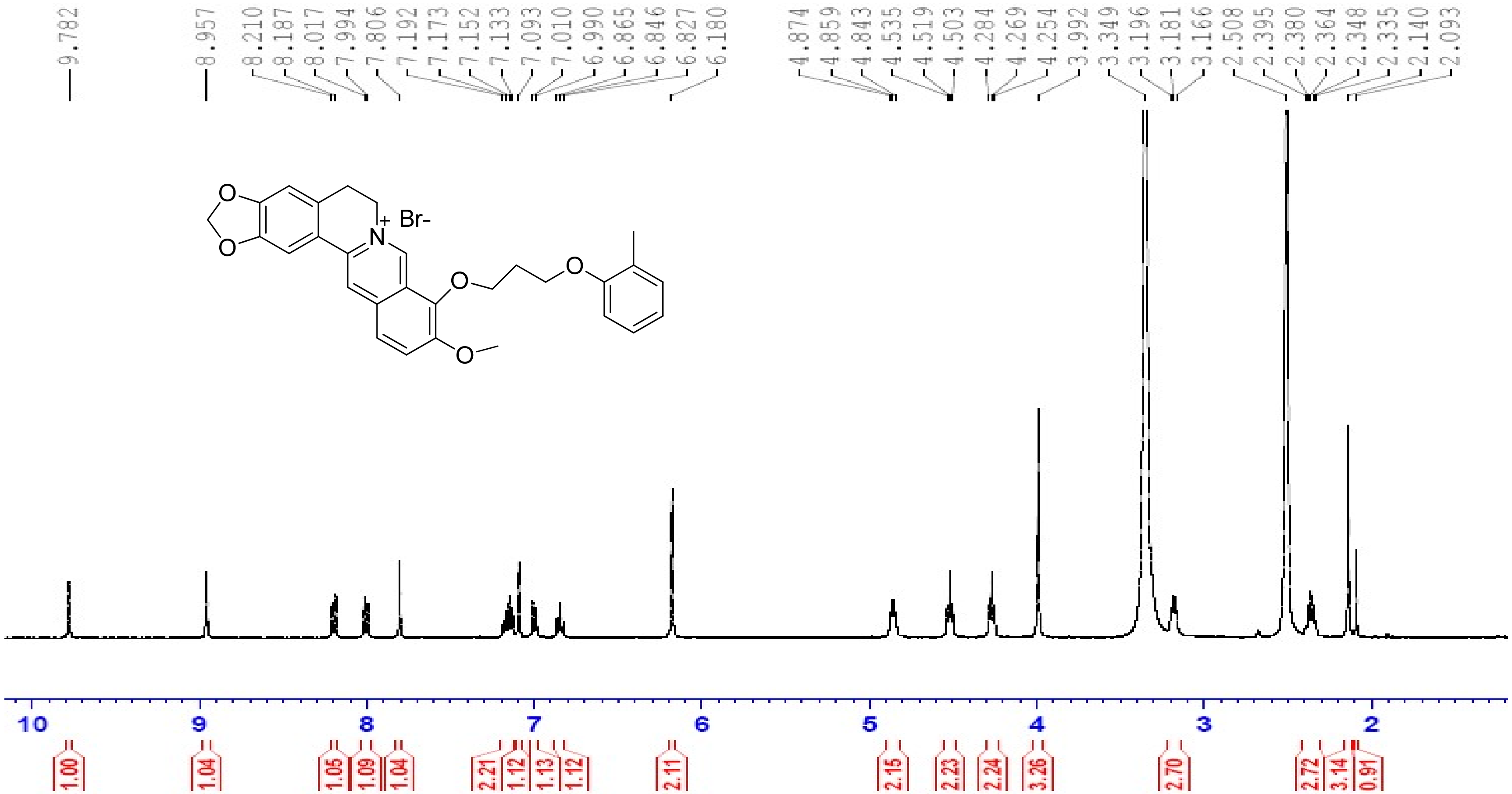

^{1}H NMR spectrum of compound **4**

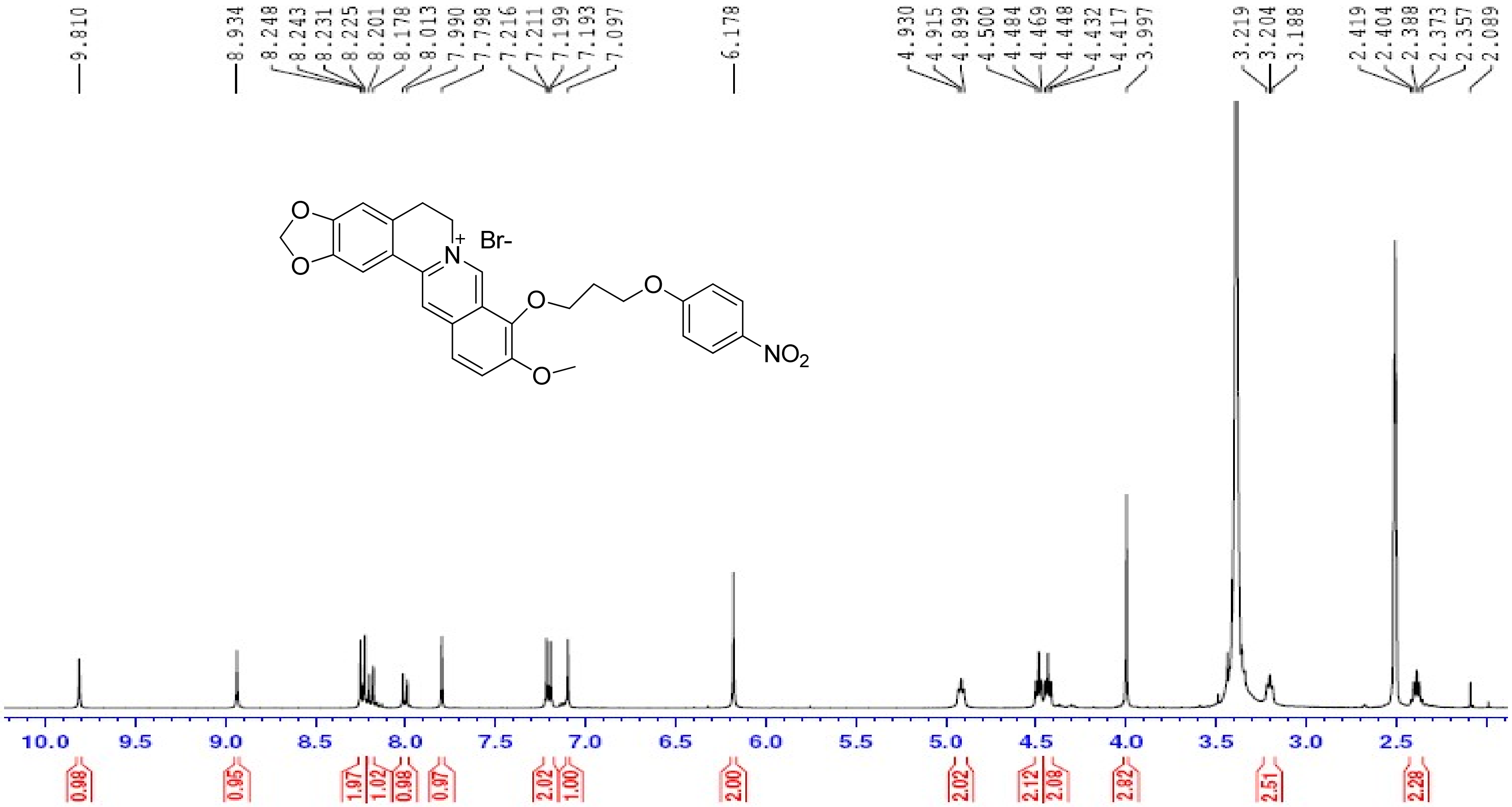

^{1}H NMR spectrum of compound **5**

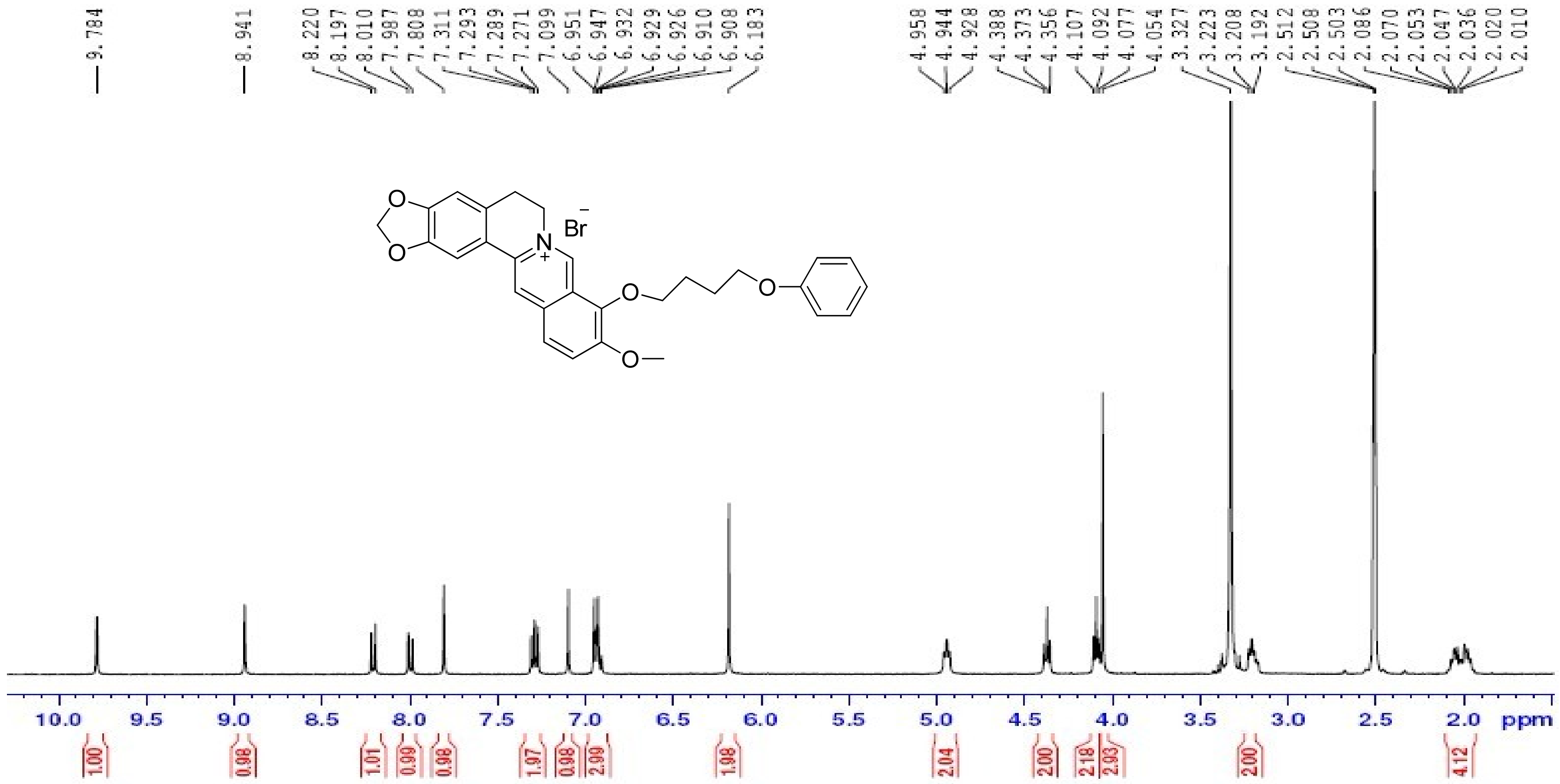

^{1}H NMR spectrum of compound **6**

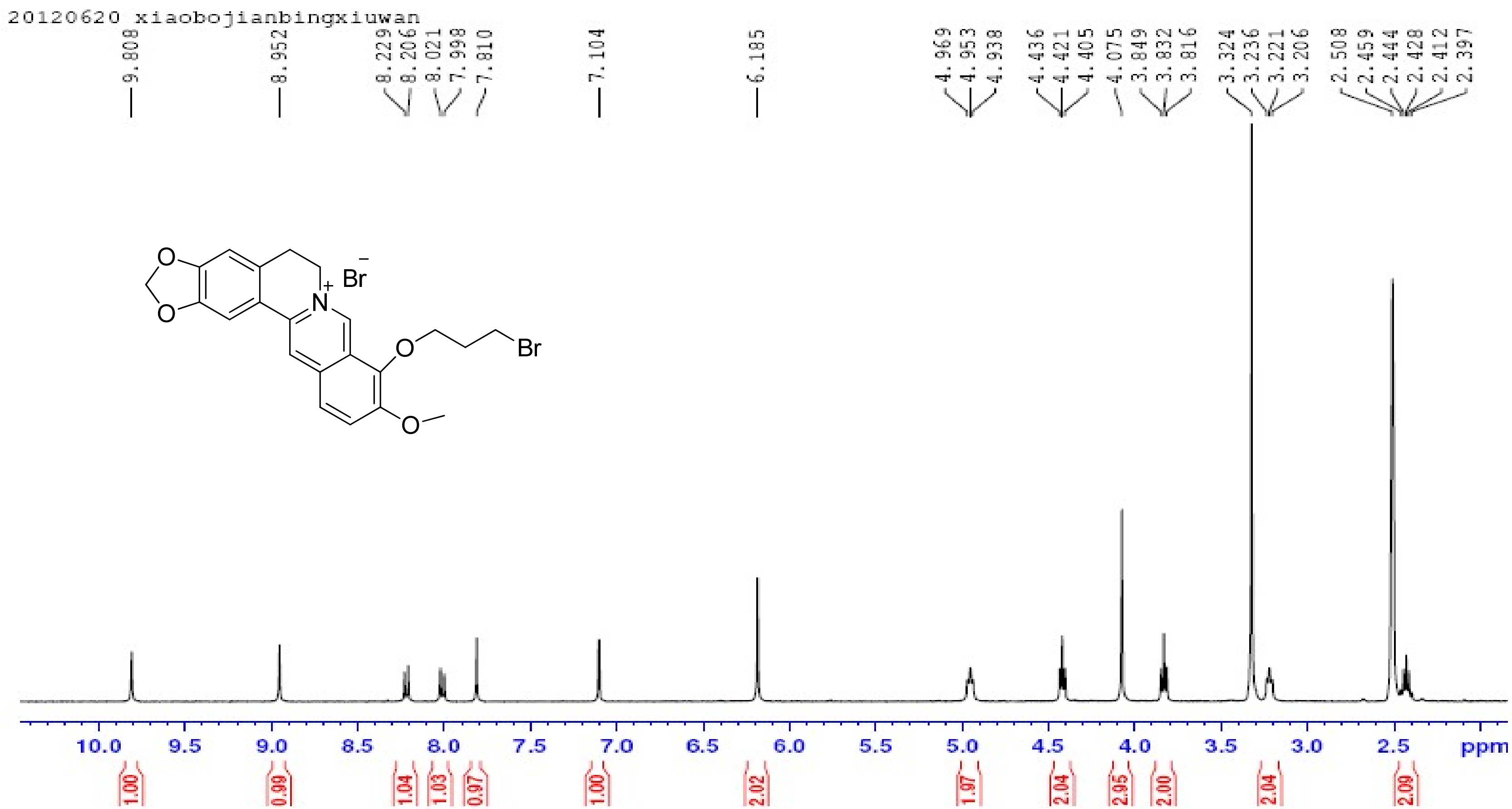

^{1}H NMR spectrum of compound **7**

References

1. Huang L, Luo Z, He F, Lu J, Li X (2010) Synthesis and biological evaluation of a new series of berberine derivatives as dual inhibitors of acetylcholinesterase and butyrylcholinesterase. Bioorganic & Medicinal Chemistry 18: 4475-4484.
2. Zhang WJ, Ou TM, Lu YJ, Huang YY, Wu WB, et al. (2007) 9-Substituted berberine derivatives as G-quadruplex stabilizing ligands in telomeric DNA. Bioorganic & Medicinal Chemistry 15: 5493-5501.
